# Phylogenetically evolutionary analysis provides insights into the genetic diversity and adaptive evolution of porcine deltacoronavirus

**DOI:** 10.1186/s12917-023-03863-2

**Published:** 2024-01-10

**Authors:** Zhenhua Guo, Qingxia Lu, Qianyue Jin, Peng Li, Guangxu Xing, Gaiping Zhang

**Affiliations:** 1 Key Laboratory of Animal Immunology of the Ministry of Agriculture, Henan Provincial Key Laboratory of Animal Immunology, Henan Academy of Agricultural Sciences, Zhengzhou, 450002 China; 2 School of Advanced Agricultural Sciences, Peking University, Beijing, China; 3 Jiangsu Co-innovation Center for Prevention and Control of Important Animal Infectious Diseases and Zoonoses, Yangzhou, China; 4 Vet Diagnostic & Production Animal Medicine, Iowa State University, Ames, IA USA

**Keywords:** Porcine deltacoronavirus, Genetic diversity, Recombination, Adaptive evolution, Spike protein

## Abstract

**Background:**

Porcine deltacoronavirus (PDCoV) is one of the emerging swine enteric coronaviruses (SECoVs), which has been widely prevalent in the North America and Asia. In addition to causing severe diarrhea in piglets, PDCoV also shows the potential to infect diverse host species, including calves, chickens, turkey poults, and humans. However, the clinical pathogenicity and genetic evolution of PDCoV is still not fully understood.

**Results:**

Here, we recorded an outbreak of a novel recombinant PDCoV strain (CHN-HeN06-2022) in a large nursery fattening pig farm. Genomic analysis showed that the CHN-HeN06-2022 strain shared 98.3-98.7% sequence identities with the Chinese and American reference strains. To clarify the evolutionary relationships, phylogenetic analysis was performed using the PDCoV genome sequences available in the GenBank database. Based on genetic distance and geographical distribution, the phylogenetic tree clearly showed that all the PDCoV sequences could be divided into lineage 1 and lineage 2, which were further classified into sublineage 1.1 (Chinese strains), 1.2 (the North American strains), 2.1 (the Southeast Asian strains), and 2.2 (Chinese strains). Corresponding to the evolutionary tree, we found that, compared to lineage 1, lineage 2 strains usually contain a continuous 6-nt deletion in *Nsp2* and a 9-nt deletion in *Nsp*3, respectively. Furthermore, recombination analysis suggested that the CHN-HeN06-2022 occurred segments exchange crossed *Nsp2* and *Nsp3* region between sublineage 1.1 and sublineage 2.1. Combined with previously reported recombinant strains, the highest recombination frequency occurred in *Nsp2*, *Nsp3*, and *S* gene. Additionally, we identified a total of 14 amino acid sites under positive selection in spike protein, most of which are located in the regions related with the viral attachment, receptor binding, and membrane fusion.

**Conclusions:**

Taken together, our studies provide novel insights into the genetic diversity and adaptive evolution of PDCoV. It would be helpful to the development of vaccine and potential antiviral agent.

**Supplementary Information:**

The online version contains supplementary material available at 10.1186/s12917-023-03863-2.

## Background

Coronaviruses (CoVs) are enveloped singe positive-stranded RNA viruses belonging to the *Coronaviridae* family, order *Nidovirales* [1]. Based on the genetic diversity, CoVs are further classified into four genera: *Alphacoronavirus* (α-CoV), *Betacoronavirus* (β-CoV), *Gammacoronavirus* (γ-CoV), and *Deltacoronavirus* (δ-CoV) [2]. Coronaviruses (CoVs) are important viral pathogens which can cause severe respiratory, digestive, and nervous diseases in mammals and birds [1]. In pigs, the swine enteric coronaviruses (SECoVs) are the most important ones which lead to severe clinical diseases and huge economic losses. To date, four different SECoVs have been reported, including porcine epidemic diarrhea virus (PEDV), transmissible gastroenteritis coronavirus (TGEV), porcine deltacoronavirus (PDCoV), and swine acute diarrhea syndrome coronavirus (SADS-CoV) [3]. Among them, PEDV and TGEV belong to α-CoV and have been prevalent in pigs for decades, whereas the δ-CoV PDCoV and α-CoV SADS-CoV are newly emerging SECoVs in recent years [4–6]. All of them cause similar clinical symptoms including vomiting, watery diarrhea, anorexia, dehydration, weight loss, and lethargy in neonatal piglets [3, 7].

The genome size of PDCoV is about 25.4 kb. The genome organization is consistent with the other CoVs, including 5′ untranslated region (UTR), open reading frames1a/1b (ORF1a/1b), spike protein (S), envelope (E), membrane (M), nonstructural protein 6 (NS6), nucleocapsid (N), NS7, 3′ UTR, and poly (A) tail [8]. PDCoV was first reported to cause severe diarrhea in suckling piglets in the United States in 2014 [9, 10]. However, the genomic information of PDCoV had been found from porcine rectal swabs as early as 2009 in Hong Kong (reported in 2012) [6]. Retrospective studies revealed that PDCoV was detectable in clinical samples collected from 2004 in China [11], which suggested that PDCoV has been circulating in pigs more than ten years before its first report. Currently, PDCoV has been found widely circulating in pigs in the North America, China, East Asia and Southeast Asia [1, 2]. Although it is well known that PDCoV infection lead to severe diarrhea in suckling piglets with very high morbility and mortality, its clinical pathogenicity on pigs of other ages still needs to be further elucidated. On the other hand, recent studies showed that PDCoV also can infect calves, chickens, and turkey poults [1, 12]. Lednicky et al. reported that PDCoV was detected in plasma samples of three Haitian children with an acute undifferentiated febrile illness [13]. All these studies suggest that PDCoV has the potential to infect diverse host species, raising the possibility of cross-species transmission of δ-CoVs.

High frequency of genetic variation and recombination is a significant characteristic for the RNA viruses [2]. In PDCoV, genetic diversity based on geographical dispersion and recombination strains have been identified by several studies [2, 14, 15]. The increase of genome sequences will facilitate us to better understand the genetic evolution and molecular characteristics of PDCoV. In present study, we recorded an outbreak of PDCoV in a large nursery pig farm. The genetic evolution and molecular characterization of the PDCoV strain were also analyzed. Furthermore, we detailedly analyzed the molecular epidemiology and adaptive evolution of PDCoV based on the currently available genome sequences in the GenBank database.

## Results

### Clinical status and laboratory diagnosis

**Fig. 1:**
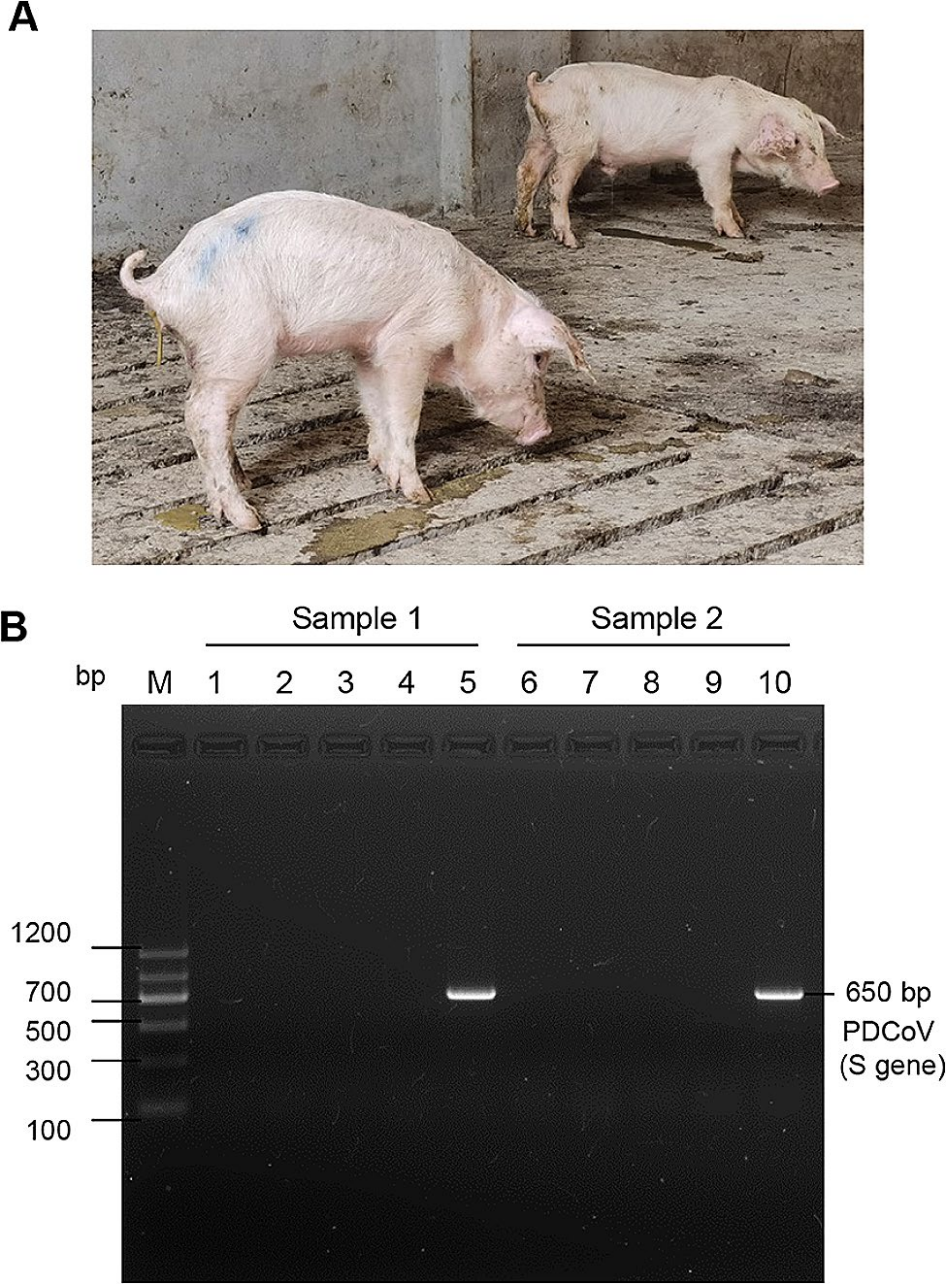
Clinical symptoms and laboratory diagnosis. (**A**) The diseased pigs displayed anorexia, vomiting, watery diarrhea, dehydration, and weight loss. (**B**) The PCR result of nucleic acids testing of the potential viral pathogens. PEDV (line 1 and 6), TGEV (line 2 and 7), PoRV (line 3 and 8), SADS-CoV (line 4 and 9), and PDCoV (line 5 and 10). The original full-length gels for the PDCoV laboratory diagnosis were presented in supplementary file 2

On April 30, 2022, severe diarrhea broke out in a commercial nursery to fattening pig farms in Henan province, central China. The farm owned approximate 7000 pigs which were purchased from a breeding company about 400 km away on April 10, 2022. The disease originally was first spotted in one barn. And the infected pigs showed syndromes of vomiting, watery diarrhea, and dehydration (Fig. 1A), which is similar with the clinical symptoms of the infection of SECoVs. To confirm the causative agent, fecal swabs were delivered to our lab and screened for enteric virus using RT-PCR assay. The test result showed that only the nucleic acid of PDCoV was positive, and that of the PEDV, TGEV, PoRV and SADS-CoV were not detected (Fig. 1B). Although farm technicians took therapeutic and biosafety measures (antibiotic treatment, regular disinfection, management of personnel and material movement), the disease eventually spread throughout the pig farm. The epidemic lasted about 105 days, and a total of 1050 pigs were dead or culled, with loss rate of approximate 15% (1050/7000).

### Genome sequencing and homology analysis

Next, we sequenced the complete genome of the PDCoV strain and submitted to GenBank under the accession number OP501870, named CHN-HeN06-2022. The genomic length of CHN-HeN06-2022 is 25,403 nucleotides (nt) (Supplementary file 1). The genome structure is similar to previous report, with the typical gene order 5′ UTR, ORF1a/1b, S, E, M, NS6, N, NS7, 3′ UTR, and poly(A) tail. Then, the identities of CHN-HeN06-2022 nucleotide and deduced amino acid (aa) sequences were analyzed using the Clustal *W* method by the MegAlign program (Table 1). The CHN-HeN06-2022 shared 98.3–98.7% genomic sequence identities with the Chinese and American reference strains (GenBank no. KP757890, JQ065042, KP757891, and KT381613), and 97.5% similarities with the Southeast reference strain S5011/Thailand/2015 (GenBank no. KU051641), respectively. Furthermore, compared with the reference strains, the *S* gene showed the most genetic diversity, with the identities of 95.9–97.9% at nt level and 96.8–98.1% at aa level, respectively. While the *E* gene was the most conserved, with 99.2–100% (nt) and 100% (aa) similarities to the reference strains.

**Table 1:** Nucleotide and amino acid sequence similarity analysis between CHN-HeN06-2022 and the reference strains

CHN-HeN06-2022 (OP501870)	Similarity (nt/aa)				
	CHN-AH-2004/China/2004 (KP757890)	HKU15-44/China/2009 (JQ065042)	CHN-HB-2014/China/2014 (KP757891)	OH11846/USA/2014 (KT381613)	S5011/Thailand/2015 (KU051641)
Genome	98.3/–	98.4/–	98.7/–	98.5/–	97.5/–
5′ UTR	99.4/–	99.4/–	99.4/–	99.3/–	99.3/–
ORF1a	98.2/98.9	98.1/98.8	98.6/99.2	98.3/99.1	97.6/98.3
ORF1b	98.8/99.7	98.6/99.7	99.1/99.8	99.0/99.7	97.8/99.4
S	97.4/97.8	97.9/98.0	97.9/98.1	97.8/98.3	95.9/96.8
E	99.2/100.0	100.0/100.0	100.0/100.0	99.6/100.0	100.0/100.0
M	98.9/98.6	98.8/98.6	98.8/98.6	98.6/98.6	98.3/98.6
N	98.1/98.8	98.8/99.7	98.7/99.7	98.8/99.7	97.3/98.8
NS6	98.9/98.9	99.3/99.3	99.3/99.3	98.9/98.9	98.2/98.2
NS7	98.8/97.5	99.3/98.5	99.2/98.0	99.2/98.0	97.7/94.5
3′ UTR	98.4/–	98.4/–	98.4/–	98.4/–	97.4/–

### Genetic evolution and molecular characteristics of PDCoVs

**Fig. 2:**
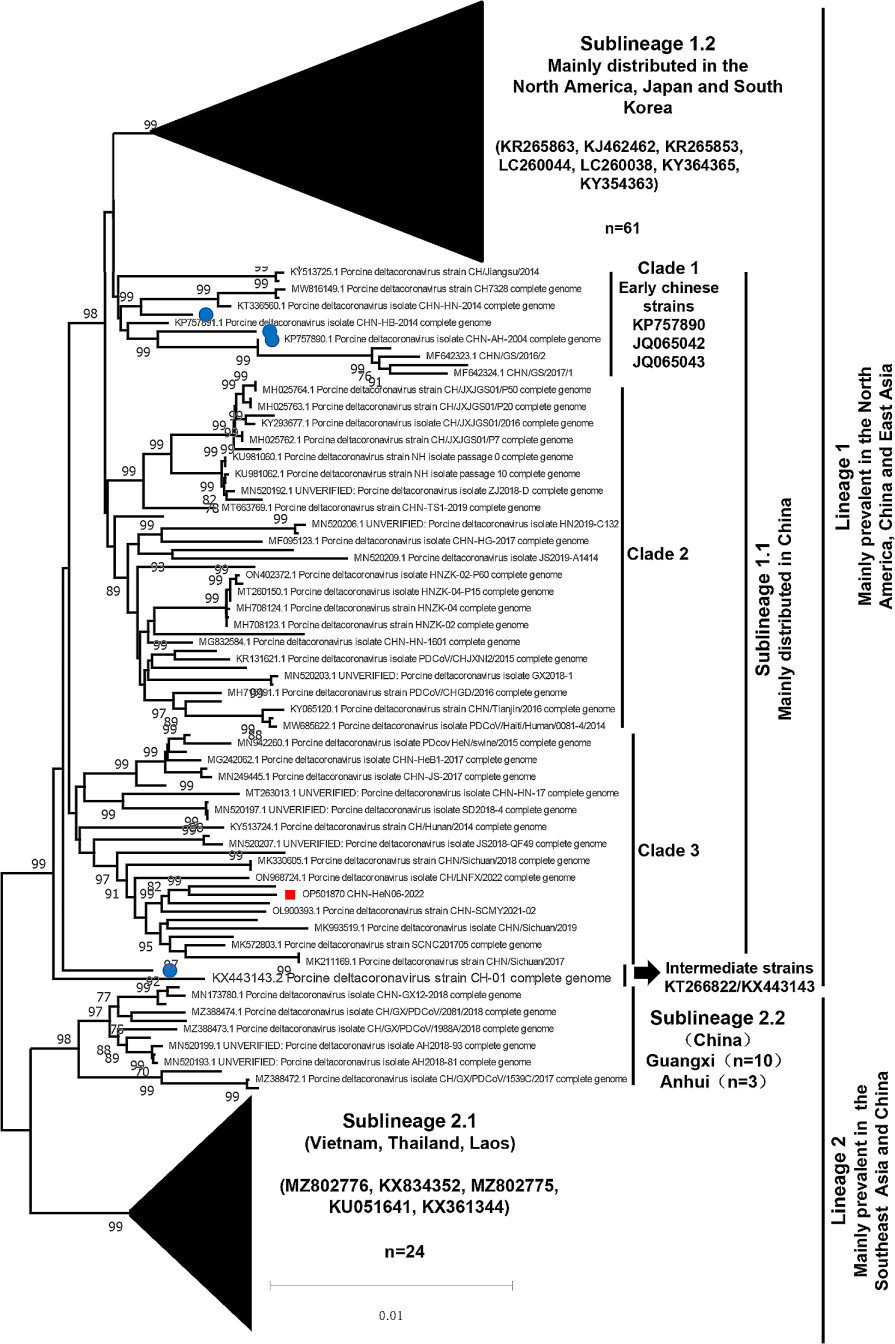
Phylogenetic analysis based on the complete genomes of PDCoVs. The isolate CHN-HeN06-2022 and other 182 genome sequences collected from GenBank (Oct. 20, 2022) were used to perform nucleotide alignment by MAFFT software. Then, the evolutionary analyses were conducted in MEGA11 using the neighbor-joining method and the Kimura-2-parameter nucleotide substitution model with 1000 bootstrap replicates. The red square symbol indicates the CHN-HeN06-2022 strain. The blue dots represent the early strains in China

To better understand the evolutionary relationship of PDCoVs, the isolate and the other 182 genome sequences (Table S1) collected from GenBank (Oct. 20, 2022) were firstly aligned by MAFFT. And then the phylogenetic analysis was performed in MEGA11.0. As shown in Fig. 2, the phylogenetic tree showed that all the PDCoVs could be clearly divided into two lineages-lineage 1 and lineage 2. Lineage 1 contains the strains mainly prevalent in the North America, China and East Asia. Lineage 2 strains were mainly distributed in the Southeast Asia and China. Moreover, lineage 1 was further divided into sublineage 1.1 and sublineage 1.2. The sublineage 1.1 has three evolutionary branches (clade 1, clade 2 and clade 3) and was mainly distributed in China, while sublineage 1.2 was main prevalent in the America, Japan, and South Korea. Similarly, lineage 2 was also divided into sublineage 2.1 (prevalent in Vietnam, Thailand, and Laos) and sublineage 2.2 (only reported in Guangxi and Anhui provinces in China) (Fig. 2). Our data indicate that genetic diversity in PDCoV is geographically distributed.

**Fig. 3:**
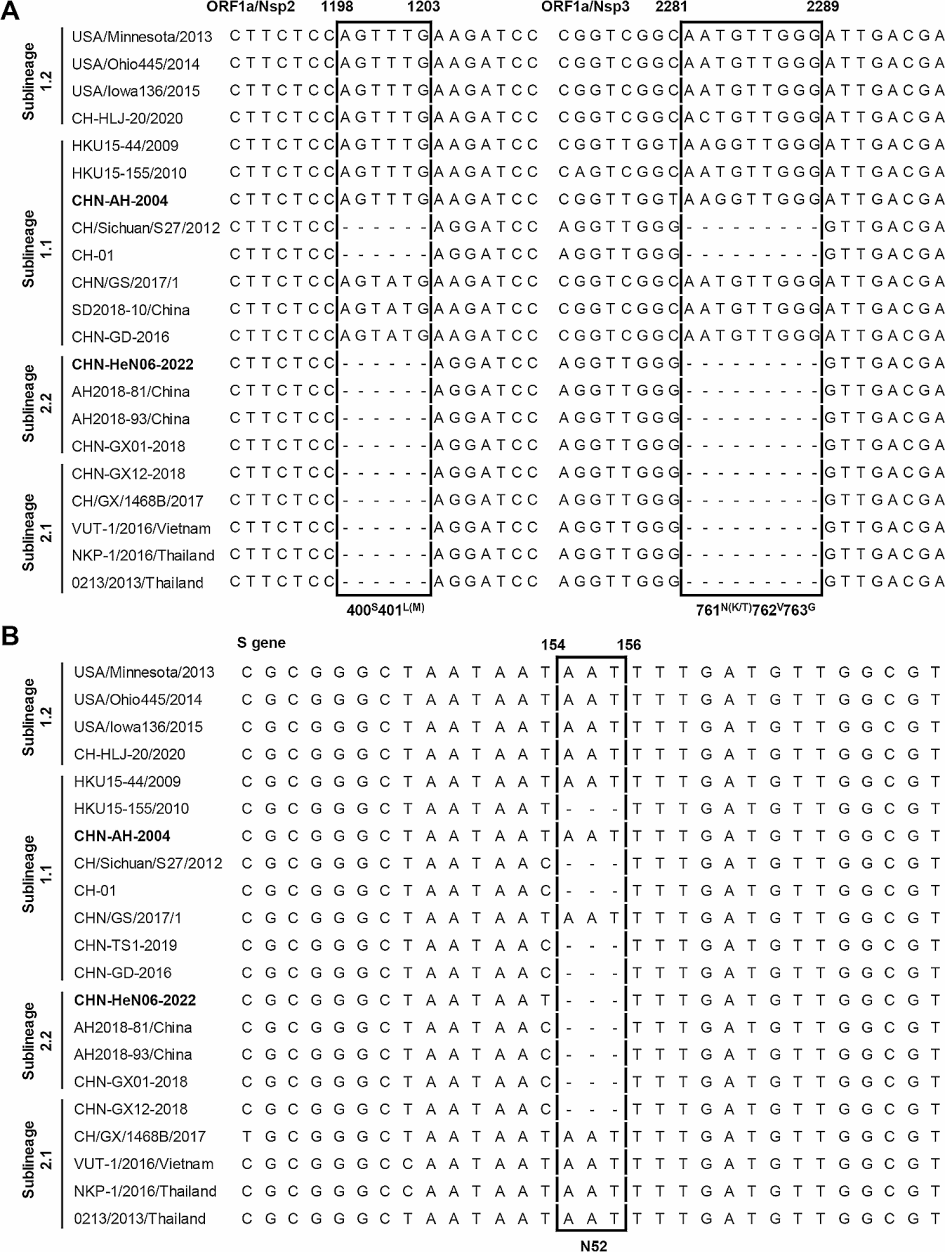
The insertion and deletion patterns of PDCoVs. The multiple sequence alignment was analyzed by the clustal *W* (codons) method in MEGA11. The earliest strain CHN-AH-2004 and the isolate CHN-HeN06-2022 were shown in bold. Using the CHN-AH-2004 strain as prototype, the insertion and deletion characteristics were displayed in *Nsp2*, *Nsp3* (**A**), and *S* gene (**B**)

Additionally, we also compared the molecular characteristics among different phylogenetic branches (representative sequences alignment were shown in Fig. 3). Using the earliest strain CHN-AH-2004 (GenBank no. KP757890) as the prototype, most of sublineage 1.1 and sublineage 2.2 strains (Chinese strains) have 1 aa deletion at the 52th position of *S* gene, while no such deletion was found in most of sublineage 1.2 strains (the North American and East Asia strains) and sublineage 2.1 strains (the Southeast Asia strains). On the other hand, lineage 2 always has a deletion pattern comprised of 6-nt deletion at positions 1,198–1,203 in *Nsp2* and 9-nt deletion at positions 2,281–2,289 in *Nsp3*, which are rarely found in lineage 1 except of the recombinant strains that exchanged gene segments with the Southeast Asia strains. Besides, we also found the CH/Sichuan/S27/2012 (GenBank no. KT266822) and CH-01 (GenBank no. KX443143) may be as the intermediate strains between lineage 1 and lineage 2 (Fig. 2), which has a deletion pattern containing 6-nt deletion in *Nsp2*, 9-nt deletion in *Nsp3*, and 3-nt deletion in *S* gene (Fig. 3). Interestingly, although the CHN-HeN06-2022 was clustered into clade 3 in sublineage 1.1 (Fig. 2), the deletion pattern of *Nsp2* and *Nsp3* in CHN-HeN06-2022 was completely consistent with the representative sequences of sublineage 2.1 and 2.2 (Fig. 3), which suggests that possibly there occurred a recombinant event in the CHN-HeN06-2022.

### Recombination analysis

**Fig. 4:**
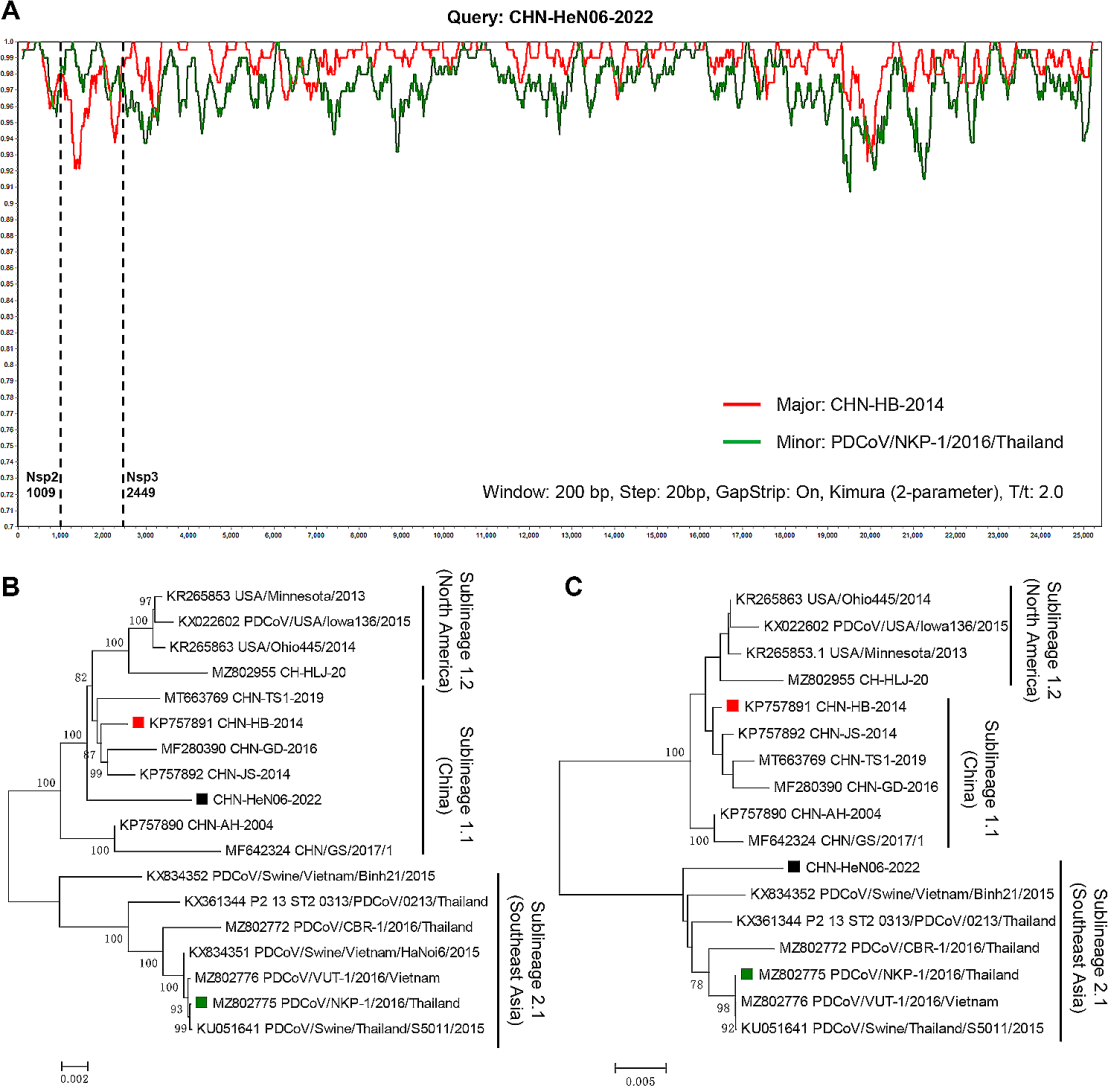
Recombination analysis of CHN-HeN06-2022. (**A**) The mosaic regions were verified by SimPlot 3.5.1 software. The *X-axis* shows the location of the query strain CHN-HeN06-2022, and the *Y-axis* displays the percentage of similarity. The major parental strain (red) and the minor parental strain (green). (**B**) and (**C**) The phylogenetic trees were constructed based on the major and minor exchanged sequences using MEGA11, respectively

**Fig. 5:**
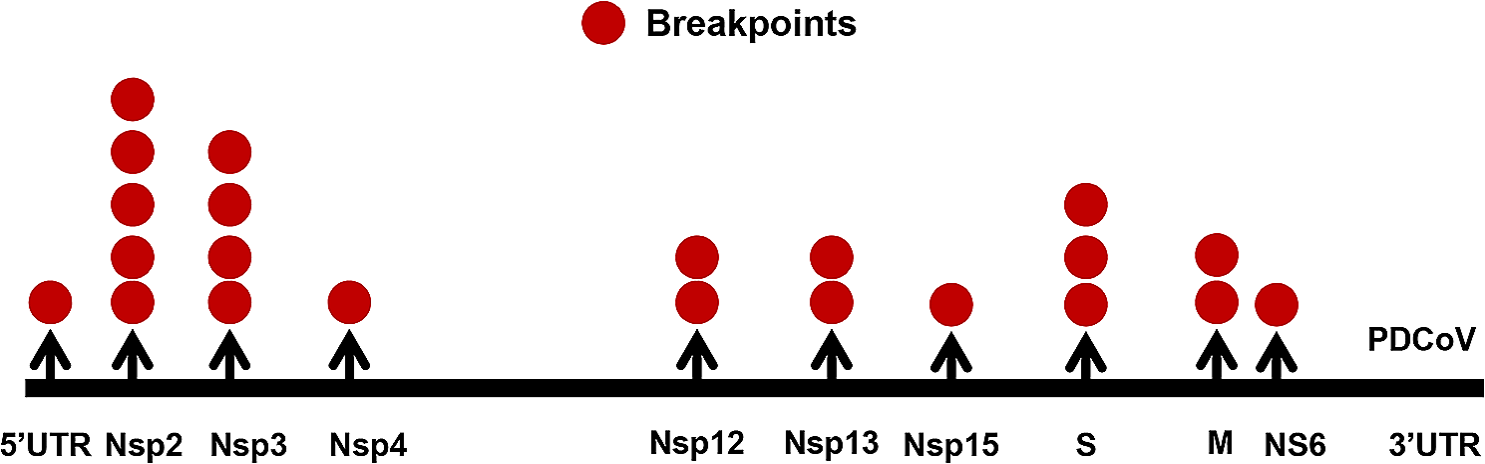
The distribution of recombinant breakpoints in the PDCoV genome. All the reported recombinant breakpoints (Oct. 20, 2022) including CHN-HeN06-2022 in this study were exhibited in the PDCoV genome

To explore the potential recombinant event in CHN-HeN06-2022, we performed a screening by RDP4.0 using seven algorithms. The statistical results suggested that CHN-HeN06-2022 was a recombinant strain between the sublineage 1.1 (Chinese strains) and sublineage 2.1 (Southeast Asian strains) (*P* < 0.001, recombinant score = 0.65), which used CHN-HB-2014 (GenBank no. KP757891) as the major parent and NKP-1/2016/Thailand (GenBank no. MZ802775) as the minor parent (Table 2). The recombinant event was also confirmed by SimPlot 3.5.1. The similarity plot showed that the potential breakpoints in CHN-HeN06-2022 to be at nt 1009 and 2449, which crossed the *Nsp2* and *Nsp3* genes (Fig. 4A). Moreover, phylogenetic analysis based on the major or minor parental sequences further indicated that the recombination event had occurred between the sublineage 1.1 and sublineage 2.1 (Fig. 4B, C). Next, we wonder whether there are recombination hotspots over the PDCoV genome. As shown in Table 3 and Fig. 5, all the reported recombinant PDCoV strains were summarized and analyzed [14, 16–22]. The data displayed that the recombinant events often occurred in *Nsp2* (5 times) and *Nsp3* (4 times), followed by *S* gene (3 times), *Nsp12* (2 times), *Nsp13* (2 times), *M* gene (2 times), 5′ UTR (1 time), *Nsp4* (1time), *Nsp15* (1 time), and *NS6* (1 time). We also found that most of the recombinant strains usually exhibited gene segments exchange between Chinese strains (sublineage 1.1) and the Southeast Asian strains (sublineage 2.1). There are still no recombination reports in the North American strains (sublineage 1.2).

**Table 2:** The recombination assay performed by RDP4.0 software

Recombinant	Major parent	Minor parent	Position	Detection methods (Av.P-Val)							Recombinant score
				RDP	GENECONV	BootScan	MaxChi	Chimaera	SiScan	3Seq	
CHN-HeN06-2022	CHN-HB-2014	NKP-1/2016/Thailand	1009–2449	8.71 × 10^− 14^	1.52 × 10^− 6^	1.05 × 10^− 8^	2.60 × 10^− 4^	4.60 × 10^− 5^	7.15 × 10^− 4^	NS	0.65

NS: not significant

**Table 3:** The recombinant PDCoV strains and the recombination regions

Strain name	Major parent	Minor parent	Breakpoints	References
CHN-GX01-2018	CHN-HB-2014	Vietnam/Binh21/2015	*Nsp2* (124)-*Nsp3* (3447); *Nsp12* (12,843)-*Nsp13* (14,847)	Huang et al. Vet Med Sci. 2020
CHN-GX09-2018				
CHN-GX11-2018				
CHN-GX12-2018				
CHN-GX81-2018	Vietnam/Binh21/2015	CHN-HB-2014	*Nsp2* (3465)-*Nsp12* (12,843); *Nsp13* (15,406)-*Nsp15* (17,631)	
CHN-HG-2017	CH/SXD1/2015	Vietnam/HaNoi6/2015	*Nsp3* (3153–4249)	Zhang et al. Arch Virol. 2018
CHN-SC2015	SHJS/SL/2016	TT-1115	*Nsp3* (6020)-Nsp4 (7069)	Zhao et al. Viruses. 2019
CHN/CQ/BN23/2016	CHN-AH-2004	Thailand/S5015L/2015	5′ UTR (471)-*Nsp2* (1442)	Wang et al. Arch Virol. 2022
P29_15_VN_1215	P12_14_VN_0814	USA/Minnesota159/2014	*S* (19,454)-*M* (23,512)	Saeng-chuto et al. Transbound Emerg Dis. 2020
P30_15_VN_1215				
P1_16_VN_0116				
Binh21	S5011	HKU15-44	*Nsp2* (1007)-*NS6* (23,846)	Le et al. Arch Virol. 2018
HaNoi6	S5011	HKU15-44		
CHN-GD16-03	CHN-JS-2014	P1_16_BTL_0115	*S* (19,251)-*M* (23,074)	Mai et al. Genome Announcements 2017
CH-HA2–2017	CH-HA3–2017	HKU15–155	*S* (27-1234)	Zhang et al. Infect Genet Evol. 2019
CHN-HeN06-2022	CHN-HB-2014	PDCoV/NKP-1/2016 /Thailand	*Nsp2* (1009)-*Nsp3* (2449)	This study

### Amino acid selection analysis of spike protein

**Fig. 6:**
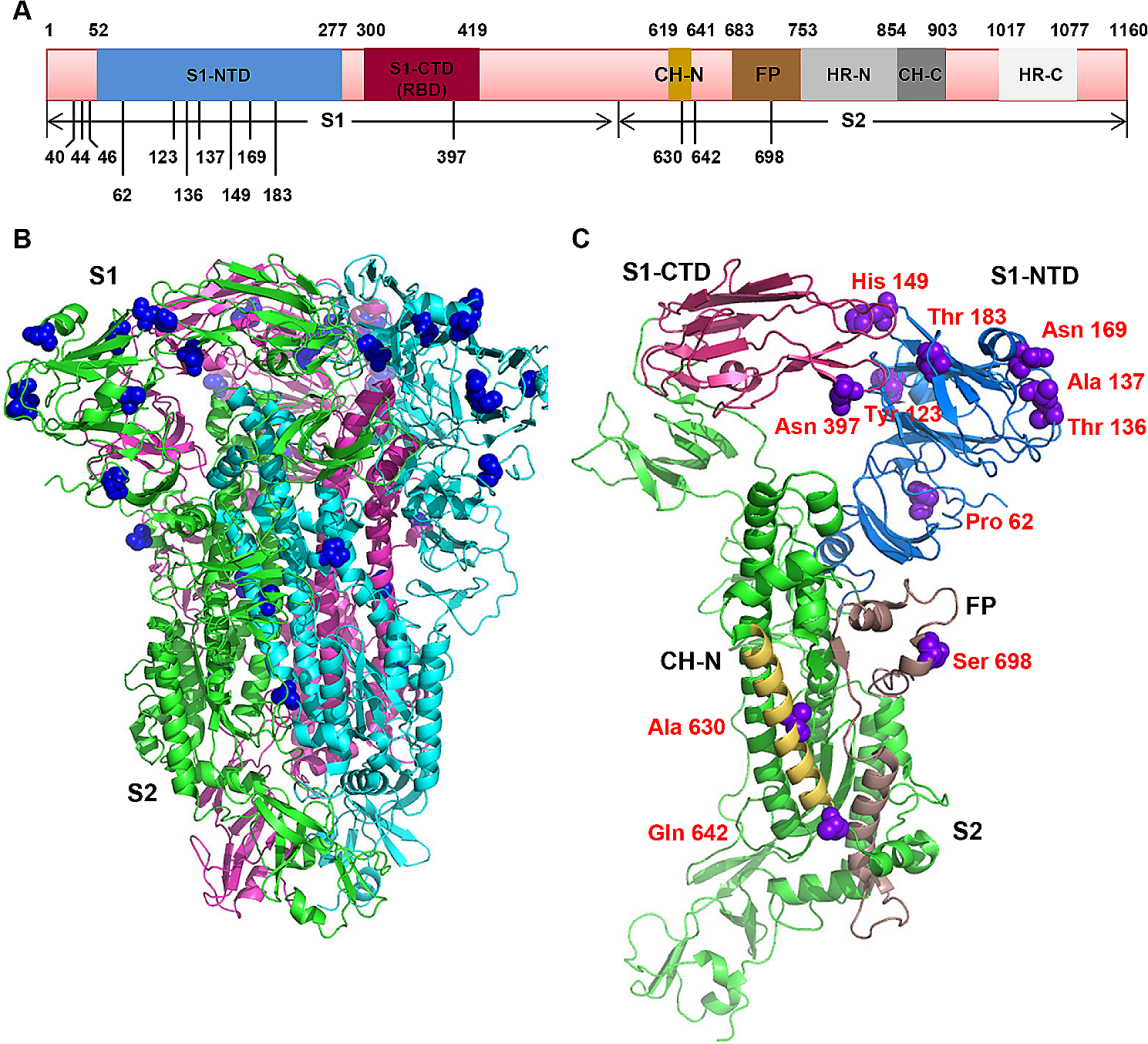
The location of the positive selection amino acid sites in spike protein. (**A**) The selection sites were showed in the schematic structure of *S* gene. The spike protein contains S1 subunit and S2 subunit. S1-NTD, N-terminal domain of S1. S1-CTD, C-terminal domain of S1. RBD, receptor binding domain. CH-N and CH-C, central helices N and C. FP, fusion peptide. HR-N and HR-C, heptad repeats N and C. (**B**) Cartoon representation of the spike homotrimer. The blue spheres indicate the selected amino acid sites and most of them are distributed on the surface. (**C**) The selected sites (purple spheres) were displayed on the structure of PDCoV S monomer using PyMOL software. The residue sites (62, 123, 136, 137, 149, 169, and 183) are in the S1-NTD. The site 397 is located in the RBD. The residue 630 and 642 are in the CH-N region and the residue 698 is the FP region. The residues 40, 44, and 46 are not resolved in the known crystallographic structure. The raw data of positive selection analysis across the spike gene was provided in supplementary file 3

The CoVs S gene is the main determinant of host tropism and related to the cross- species transmission [12]. To better understand the adaptive evolution, we investigated the positive amino acids selection in *S* gene of PDCoV using the Datamonkey (http://www.datamonkey.org/). Finally, we identified 14 aa sites under the positive selection in the *S* gene (Table 4; Fig. 6A). Among them, 11 sites (residues 40, 44, 46, 62, 123, 136, 137, 149, 169, 183, and 397) were distributed in S1 subunit, which is mainly responsible for receptor binding and immune response [23]. While 3 sites (residues 630, 642, and 968) were located in the S2 subunit, which is mainly responsible for membrane fusion [23]. Besides, the residues 149 had the highest variability among the PDCoVs, followed by residues 44 and 169.

**Table 4:** Amino acid selection analysis of the PDCoV S protein

No.	Site	FUBAR (Post.pro)	FEL (P value)	MEME (*P* value)	Amino acid
1	40	0.971	0.0554	0.08	Ser
2	44	0.988	0.0259	0.04	Ser
3	46	0.974	0.0361	0.05	Tyr
4	62	0.976	0.0259	0.04	Pro
5	123	0.989	0.0194	0.03	Tyr
6	136	0.98	0.0279	0.04	Thr
7	137	0.986	0.0205	0.03	Ala
8	149	0.998	0.0053	0.01	His
9	169	0.998	0.0032	0.01	Asn
10	183	0.981	0.0271	0.04	Thr
11	397	0.979	0.0428	0.06	Asn
12	630	0.961	0.029	0.01	Ala
13	642	0.992	0.0433	0.05	Gln
14	698	0.954	0.0398	0.06	Ser

Then, the positive selective aa sites were further visualized on a three-dimensional cartoon diagram using the cryo-electron microscopy (cryo-EM) structure of PDCoV S-e (residues 52-1017) (Fig. 6B, C) [23]. Specifically, the residues 62, 123, 136, 137, 149, 169, and 183 are on the surface of homotrimer. And all of them are located on the N-terminal domain of S1 (S1-NTD) which may facilitate the initial viral attachment to cells. The residue 397 is inner of the trimeric structure and mapped in the receptor binding domain (RBD) that may alter the receptor specificity and consequently tissue tropism. The residue 630 and 642 are located in the central helices N (CH-N) region consisting of four helices and a connecting loop. And the residue 698 is distributed in the fusion peptide (FP) region which can insert into the target membrane after a conformation change. On the other hand, the residues 40, 44, and 46 are not resolved in the known crystallographic structure, possibly due to the fact that the fragment is too flexible to be seen by cryo-EM.

## Discussion

It is well known that PDCoV causes severe diarrhea in suckling piglets. However, there are very few clinical reports on the pathogenicity of PDCoV in nursery pigs. Here, we recorded an outbreak of PDCoV in a large nursery pig farm which raised about 7000 pigs. The diseases lasted more than 3 months and almost all of the pigs were infected. Finally, approximate 1050 pigs were dead or culled. The direct loss rate was about 15%. More importantly, the growth performance of most infected pigs was severely affected, manifesting obvious wasting and growth retardation. Our detailed document will provide valuable information for the clinical prevention and control strategies of PDCoV. Given the growing epidemic of PDCoV and the huge economic loss, it is urgent to develop effective vaccines or drugs for prevention and treatment.

The PDCoV strain was successfully sequenced and named CHN-HeN06-2022. Homological analysis showed that the CHN-HeN06-2022 have the highest nt identity with the reference strain CHN-HB-2014 (GenBank no. KP757891). Specifically to the ORFs, the S gene exhibited the most genetic diversity and the E gene had the highest conservation, which are consistent with previous reports [10]. To clarify the evolutionary relationship, the CHN-HeN06-2022 and other 182 genome sequences of PDCoVs were used to perform phylogenetic analysis. The results indicated that all of the PDCoVs could be divided into two lineages-lineage 1 and lineage 2, which were further splitted into sublineage 1.1 (Chinese strains), sublineage 1.2 (the North American strains), sublineage 2.1 (the Southeast Asian strains), and sublineage 2.2 (Chinese strains). Based on the geographical distribution of PDCoV strains, previous studies divided the PDCoV full genomes into three major lineages, including the Southeast Asia (SEA) lineage, America lineage, and China (CHN) lineage [1, 2, 7]. However, the classification can’t fully reflect the genetic evolution of the PDCoV strains. In this study, by combining genetic distance and geographical distribution, our research showed that the PDCoVs could be clearly divided into two lineages-lineage 1(Mainly prevalent in the North America, China and East Asia) and lineage 2 (Mainly prevalent in the Southeast Asia and China), which provides a new perspective to understand the evolutionary relationships of PDCoVs. In China, most PDCoV strains were clustered into sublineage 1.1, with a small number of them belonging to sublineage 2.2, and a very few classified into sublineage 1.2 or sublineage 2.1. Given that China is the largest consumer of pork and maintains frequent pig industry-related trades with other major pig raising countries, it is not surprising that PDCoV strains are so complicated in China. For example, the PDCoV strains belonging to sublineage 2.2 were mainly from Guangxi province, which is adjacent to Vietnam. It is reasonable to speculate that the sublineage 2.1 strains were introduced into Guangxi from Vietnam and went through locally genetic evolution or recombination to form a novel phylogenetic branch.

Several studies have reported that, in some PDCoV strains, continuous 3-nt, 6-nt, or 9-nt deletion could occur in *S* gene, *Nsp2* gene, or *Nsp3* gene, respectively [14, 21, 22]. However, it is not clear whether this insertion or deletion pattern is associated with the PDCoVs classification. Here, we confirmed that, corresponding to the genetic evolution, specific insretion or deletion pattern could be as molecular features in different lineages. Briefly, compared to lineage 1, lineage 2 strains usually contain continuous 6-nt deletion in *Nsp2* and 9-nt deletion in *Nsp*3. On the other hand, the *S* gene of most Chinese strains has 1 aa deletion at the 52th position, which are rarely found in the North American strains (sublineage 1.2) and Southeast Asian strains (sublineage 2.1). Furthermore, it seems that the specific insertion or deletion pattern is not related with the pathogenicity of PDCoV, since the PDCoV strains from different sublineages displayed similar pathogenicity to newborn piglets [16–19, 24, 25]. Interestingly, although the CHN-HeN06-2022 was clustered into sublineage 1.1, it has the same deletion pattern with the sublineage 2.2 strains, suggesting that there may be a potential recombinant event. Indeed, we confirmed that the CHN-HeN06-2022 was a recombinant strain between CHN-HB-2014 (major parent) and NKP-1/2016/Thailand (minor parent). The breakpoint region crossed *Nsp2* and *Nsp3*, which make it obtain the specific deletion pattern in sublineage 2.2 strains. Combined with the previous reported recombinant strains, we found that the *Nsp2*, *Nsp3*, and *S* gene were the regions with the highest recombination frequency.

The *S* gene of CoVs usually acts as the major determinant of host cell tropism and the mediator of viral entry into host cells [26]. Thus, we further analyzed the codon sites under positive selection of *S* gene, which might have played an important role in the adaptation of PDCoV to swine. Finally, we identified a total of 14 aa sites under the positive selection. Interestingly, seven residues (sites 62, 123, 136, 137, 149, 169, and 183) are distributed on the surface of spike trimer in the S1-NTD region, which attaches to the cellular carbohydrates to keep the virus in close proximity to the host cell surface [23, 26]. One residue (site 397) is located in the RBD (S1-CTD) and buried inner of homotrimer. As we know, it is a critical step that the RBD binds to host cell receptors for the CoVs infection [27]. Thus, the aa positive selection in this region might facilitate the infection of PDCoV to the porcine cells. Besides, we also identified that the residue site 698 is located the FP region, which is responsible for the membrane fusion and finally leads to the delivery of the viral genome to the cytosol [23]. Interestingly, there are no obvious association between positive selection aa sites and PDCoV sublineages. It will be important to illustrate whether these positive selection aa sites could result in advantageous infection and replication capability of PDCoV in the future. Taken together, our results demonstrates that most of the positive selection sites are distributed on the surface of the spike trimer and located in the regions related with the viral attachment, receptor binding, and membrane fusion. These sites might be playing a positive role during the viral adaptive evolution.

Currently, PDCoVs have been widely prevalent in the major pig breeding regions and become one of the most important SECoVs to the pig industry. Here, we provided novel insights to better understand the clinical pathogenicity, evolutionary characteristics, and adaptive evolution of PDCoV. In the future, it will be valuable to explore the pathogenicity differences and immune cross-protection among the different PDCoV lineages. And it is also urgent to develop efficient vaccine and antiviral agent.

## Materials and methods

### Clinical samples collection and treatment

In April 2022, a severe diarrhea broke out in a large nursery-fattening pig farm, which is located in Xinxiang, Henan province, China and named Xingwang Farming Co., LTD. The clinical manifestations strongly supported the potential infection of enteric viral infection. To confirm the causative agent, 6 fecal swabs were collected and submitted to our lab. The fecal swabs were placed into 2 tubes (3 were mixed into 1). Then, 1 mL 1 × phosphate buffer saline (PBS) was added to each tube and went through suspension by vortex mixer. After centrifugation at 5,000 rpm for 5 min, the supernatants were collected and stored in −40 °C for the following study.

### Nucleic acid extraction and specific RT-PCR detection

The nucleic acids of samples were extracted using the FastPure Viral DNA/RNA Mini Kit (Nanjing Vazyme Biotech Co., Ltd.) according to manufacturer’s instruction. Then, the cDNA was generated by the PrimeScript RT Master Mix Kit (TaKaRa Biotechnology Co., Ltd.). To confirm the potential viral agent, the specific detection primers against the PEDV, TGEV, porcine rotavirus (PoRV), PDCoV, and SADS-CoV were used for the PCR test (Table 5) [4, 5, 28]. All the primers were synthesized by Sangon Biotech (Shanghai) Co., Ltd. For the PCR assay, a total of 25µL reaction system was used, which contains 2µL of cDNA templates, 1µL of primer pairs (2.5µM), 12.5µL 2 × Taq Plus PCR PreMix reagent (TIANGEN Biotech Co., Ltd., China). The PCR procedure was as follow: one cycle at 95 °C for 3 min; 30 cycles at 95 °C for 30 s, 55 °C for 30 s and 72 °C for 45 s, followed by elongation at 72 °C for 5 min and held at 16 °C. The products were visualized and imaged by 1.50% agarose gel electrophoresis and ultraviolet light.

**Table 5:** Detection primers used in this study

Primer name	Sequence 5′–3′	Product size (bp)	Resource
PEDV-S-For	TTCTGAGTCATGAACAGCCA	650	Dae S. Song et al., 2006
PEDV-S-Rev	CATATGCAGCCTGCTCTGAA		
TGEV-S-For	GTGGTTTTGGTYRTAAATGC	858	
TGEV-S-Rev	CACTAACCAACGTGGARCTA		
PoRV-VP6-For	AAAGATGCTAGGGACAAAATTG	308	
PoRV-VP6-Rev	TTCAGATTGTGGAGCTATTCCA		
PDCoV-N-For	TTTCAGGTGCTCAAAGCTCA	694	Leyi Wang et al., 2014
PDCoV-N-Rev	GCGAAAAGCATTTCCTGAAC		
SADS-CoV-RdRp-For	TTTTGGTTCTTACGGGCTGTT	753	Xinliang Fu et al., 2018
SADS-CoV-RdRp-Rev	CAAACTGTACGCTGGTCAACT		

### Genome sequencing

To sequence the complete genome of PDCoV strain, a total of 25 primer pairs were designed based on the reference strain CHN-HeB-A1 (GenBank no. MG242062) (Table S2). The amplicons were purified by SanPrep Column PCR Product Purification Kit and sequenced using the Sanger method (Sangon Biotech Co., Ltd.). Then, the complete genome sequence of PDCoV was obtained by sequence alignment and splicing using the DNASTAR software.

### Homology, sequence alignment, and phylogeny analysis

Several bioinformatics methods were used to investigate the genetic characteristics and evolution of the PDCoV strain. Briefly, the homology of nucleotide and deduced amino acid (aa) sequences were analyzed using clustal *W* method by the MegAlign program (DNASTAR, Inc., Madison, WI, USA). The multiple sequence alignment was performed by the clustal *W* (codons) method through Molecular Evolutionary Genetics Analysis (MEGA 11.0) to determine the genetic variations [29]. To conduct the evolutionary analysis, numerous PDCoV genome sequences were firstly went through alignment using the Multiple Alignment using Fast Fourier Transform (MAFFT) software [30]. Then, the phylogenetic tree was constructed using neighbor-joining method with Kimura 2-parameter model in MEGA 11.0 software [29].

### Recombination analysis

For recombinant analysis, all the genome alignment sequences were screened by recombination detection program 4 (RDP 4.0) using seven methods including RDP, GENECONV, BootScan, Maxchi, Chimaera, Siscan and 3Seq [31]. The recombinant events were considered to be occurred if at least six of the seven methods have a *p* value cut-off of 0.05. Then, the putative segment exchanges were further verified using Simplot 3.5.1 software to scan the genomic sequences, with a sliding window of 200 bp (20 bp step size) [32].

### Amino acid sites selection analysis of spike protein

To explore the evolutionary selection, we further inferred the positive selection of aa sites on the spike protein using the Datamonkey (http://www.datamonkey.org/). The methods to analyze the codon sites under the positive selection included Fast Unconstrained Bayesian AppRoximation (FUBAR), Fixed Effects Likelihood (FEL), and Mixed Effects Model of Evolution (MEME) [2, 33]. The aa sites were recognized as positive selection when they were identified by all these three algorithms. The spike protein models were constructed using the cryo-electron microscopy structure of PDCoV S-e (PDB ID. 6B7N) by PyMol 2.5 software (the PyMOL Molecular Graphics System, Version 2.0 Schrödinger, LLC. https://pymol.org/2/). The exhibition of the selection sites were completed with the wizard tool of the program.

### Electronic supplementary material

Below is the link to the electronic supplementary material.

**Supplementary Material 1: Table S1.** Sequence information in this study

**Supplementary Material 2: Table S2.** Primers used for genome sequencing

**Supplementary Material 3: Supplementary file 1.** The genome sequence of PDCoV strain CHN-HeN06-2022

**Supplementary Material 4: Supplementary file 2.** The original full-length gels for the PDCoV laboratory diagnosis

**Supplementary Material 5: Supplementary file 3.** The raw data of positive selection analysis across the spike gene

## Data Availability

All data generated or analyzed during this study are included in this published article and its supplementary information files. The genome sequence of CHN-HeN06-2022 (GenBank no. OP501870) is available in the website https://www.ncbi.nlm.nih.gov/nuccore/OP501870.1/.

## Abbreviations

CoVs: Coronaviruses
SECoVs: Swine enteric coronaviruses
PEDV: Porcine epidemic diarrhea virus
TGEV: Transmissible gastroenteritis coronavirus
PDCoV: Porcine deltacoronavirus
SADS-CoV: Swine acute diarrhea syndrome coronavirus
PoRV: Porcine rotavirus
cryo-EM: cryo-electron microscopy
S1-NTD: N-terminal domain of S1
RBD: Receptor binding domain
CH-N: Central helices N
FP: Fusion peptide
PBS: Phosphate buffer saline
MAFFT: Multiple Alignment using Fast Fourier Transform
RDP: Recombination detection program
FUBAR: Fast Unconstrained Bayesian AppRoximation
FEL: Fixed Effects Likelihood
MEME: Mixed Effects Model of Evolution

## References

[1] Kong F, Wang Q, Kenney SP, Jung K, Vlasova AN, Saif LJ (2022). Porcine deltacoronaviruses: origin, Evolution, cross-species transmission and zoonotic potential. Pathogens.

[2] He W-T, Ji X, He W, Dellicour S, Wang S, Li G, Zhang L, Gilbert M, Zhu H, Xing G (2020). Genomic Epidemiology, evolution, and Transmission dynamics of Porcine Deltacoronavirus. Mol Biol Evol.

[3] Turlewicz-Podbielska H, Pomorska-Mól M (2021). Porcine coronaviruses: overview of the state of the art. Virol Sin.

[4] Fu X, Fang B, Liu Y, Cai M, Jun J, Ma J, Bu D, Wang L, Zhou P, Wang H (2018). Newly emerged porcine enteric alphacoronavirus in southern China: identification, origin and evolutionary history analysis. Infect Genet Evol.

[5] Wang L, Byrum B, Zhang Y (2014). Detection and genetic characterization of deltacoronavirus in pigs, Ohio, USA, 2014. Emerg Infect Dis.

[6] Woo PC, Lau SK, Lam CS, Lau CC, Tsang AK, Lau JH, Bai R, Teng JL, Tsang CC, Wang M (2012). Discovery of seven novel mammalian and avian coronaviruses in the genus deltacoronavirus supports bat coronaviruses as the gene source of alphacoronavirus and betacoronavirus and avian coronaviruses as the gene source of gammacoronavirus and deltacoronavirus. J Virol.

[7] Song D, Zhou X, Peng Q, Chen Y, Zhang F, Huang T, Zhang T, Li A, Huang D, Wu Q (2015). Newly emerged PorcineDeltacoronavirusAssociated with Diarrhoea in Swine in China: identification, prevalence and full-length genome sequence analysis. Transboun Emerg Dis.

[8] Liang Q, Li B, Zhang H, Hu H, Thrash JC. Complete genome sequences of two porcine Deltacoronavirus strains from Henan Province, China. Microbiol Resour Ann 2019, 8(10). 10.1128/MRA.01517-18 PMC6406112 30863822

[9] Wang L, Byrum B, Zhang Y (2014). Porcine coronavirus HKU15 detected in 9 US states, 2014. Emerg Infect Dis.

[10] Marthaler D, Jiang Y, Collins J, Rossow K. Complete Genome Sequence of Strain SDCV/USA/Illinois121/2014, a Porcine Deltacoronavirus from the United States. Genome Announc 2014, 2(2). 10.1128/genomeA.00218-14 PMC3983293 24723704

[11] Dong N, Fang L, Zeng S, Sun Q, Chen H, Xiao S (2015). Porcine Deltacoronavirus in Mainland China. Emerg Infect Dis.

[12] Li W, Hulswit RJG, Kenney SP, Widjaja I, Jung K, Alhamo MA, van Dieren B, van Kuppeveld FJM, Saif LJ. Bosch B-J: broad receptor engagement of an emerging global coronavirus may potentiate its diverse cross-species transmissibility. P Nati Acad Sci 2018, 115(22). 10.1073/pnas.1802879115 PMC5984533 29760102

[13] Lednicky JA, Tagliamonte MS, White SK, Elbadry MA, Alam MM, Stephenson CJ, Bonny TS, Loeb JC, Telisma T, Chavannes S et al. Emergence of porcine delta-coronavirus pathogenic infections among children in Haiti through independent zoonoses and convergent evolution. *medRxiv* 2021.

[14] Zhang Y, Cheng Y, Xing G, Yu J, Liao A, Du L, Lei J, Lian X, Zhou J, Gu J (2019). Detection and spike gene characterization in porcine deltacoronavirus in China during 2016–2018. Infect Genet Evol.

[15] Saeng-chuto K, Lorsirigool A, Temeeyasen G, Vui DT, Stott CJ, Madapong A, Tripipat T, Wegner M, Intrakamhaeng M, Chongcharoen W (2017). Different lineage of Porcine Deltacoronavirus in Thailand, Vietnam and Lao PDR in 2015. Transbound Emerg Dis.

[16] Zhao Y, Qu H, Hu J, Fu J, Chen R, Li C, Cao S, Wen Y, Wu R, Zhao Q (2019). Characterization and pathogenicity of the Porcine Deltacoronavirus isolated in Southwest China. Viruses.

[17] Zhang M-J, Liu D-J, Liu X-L, Ge X-Y, Jongkaewwattana A, He Q-G, Luo R (2018). Genomic characterization and pathogenicity of porcine deltacoronavirus strain CHN-HG-2017 from China. Arch Virol.

[18] Wang Z, Li S, Shao Y, Lu Y, Tan C, Cui Y, Ding G, Fu Y, Liu G, Chen J (2022). Genomic characterization and pathogenicity analysis of a porcine deltacoronavirus strain isolated in western China. Arch Virol.

[19] Saeng-chuto K, Jermsutjarit P, Stott CJ, Vui DT, Tantituvanont A, Nilubol D (2019). Retrospective study, full‐length genome characterization and evaluation of viral infectivity and pathogenicity of chimeric porcine deltacoronavirus detected in Vietnam. Transbound Emerg Dis.

[20] Mai K, Li D, Wu J, Wu Z, Cheng J, He L, Tang X, Zhou Z, Sun Y, Ma J. Complete genome sequences of two porcine Deltacoronavirus strains, CHN-GD16-03 and CHN-GD16-05, isolated in Southern China, 2016. Genome Announc 2018, 6(4). 10.1128/genomeA.01545-17 PMC5786690 29371364

[21] Le VP, Song S, An B-H, Park G-N, Pham NT, Le DQ, Nguyen VT, Vu TTH, Kim K-S, Choe S (2017). A novel strain of porcine deltacoronavirus in Vietnam. Arch Virol.

[22] Huang H, Yin Y, Wang W, Cao L, Sun W, Shi K, Lu H, Jin N (2020). Emergence of Thailand-like strains of porcine deltacoronavirus in Guangxi Province, China. Vet Med Sci.

[23] Shang J, Zheng Y, Yang Y, Liu C, Geng Q, Tai W, Du L, Zhou Y, Zhang W, Li F. Cryo-Electron Microscopy Structure of Porcine Deltacoronavirus Spike Protein in the Prefusion State. J Virol 2018, 92(4). 10.1128/JVI.01556-17 PMC5790952 29070693

[24] Suzuki T, Shibahara T, Imai N, Yamamoto T, Ohashi S (2018). Genetic characterization and pathogenicity of Japanese porcine deltacoronavirus. Infect Genet Evol.

[25] Wang H, Qin Y, Zhao W, Yuan T, Yang C, Mi X, Zhao P, Lu Y, Lu B, Chen Z et al. Genetic characteristics and pathogenicity of a Novel Porcine Deltacoronavirus Southeast Asia-Like strain found in China. Front Vet Sci 2021, 8. 10.3389/fvets.2021.701612 PMC8322666 34336982

[26] Wrapp D, McLellan JS. The 3.1-Angstrom Cryo-electron Microscopy structure of the Porcine Epidemic Diarrhea Virus spike protein in the Prefusion conformation. J Virol 2019, 93(23). 10.1128/JVI.00923-19 PMC6854500 31534041

[27] Zhu X, Liu S, Wang X, Luo Z, Shi Y, Wang D, Peng G, Chen H, Fang L, Xiao S (2018). Contribution of porcine aminopeptidase N to porcine deltacoronavirus Infection. Emerg Microbes Infec.

[28] Song DS, Kang BK, Oh JS, Ha GW, Yang JS, Moon HJ, Jang YS, Park BK (2006). Multiplex reverse transcription-PCR for rapid differential detection of porcine epidemic diarrhea virus, transmissible gastroenteritis virus, and porcine group a rotavirus. J Vet Diagn Invest.

[29] Tamura K, Stecher G, Kumar S (2021). MEGA11: Molecular Evolutionary Genetics Analysis Version 11. Mol Biol Evol.

[30] Nakamura T, Yamada KD, Tomii K, Katoh K (2018). Parallelization of MAFFT for large-scale multiple sequence alignments. Bioinformatics.

[31] Martin DP, Murrell B, Golden M, Khoosal A, Muhire B (2015). RDP4: detection and analysis of recombination patterns in virus genomes. Virus Evol.

[32] Lole KS, Bollinger RC, Paranjape RS, Gadkari D, Kulkarni SS, Novak NG, Ingersoll R, Sheppard HW, Ray SC (1999). Full-length human immunodeficiency virus type 1 genomes from subtype C-infected seroconverters in India, with evidence of intersubtype recombination. J Virol.

[33] Murrell B, Moola S, Mabona A, Weighill T, Sheward D, Kosakovsky Pond SL, Scheffler K (2013). FUBAR: a fast, unconstrained bayesian AppRoximation for Inferring Selection. Mol Biol Evol.

