# Supplementary material for: Phylogenetically evolutionary analysis provides insights into the genetic diversity and adaptive evolution of porcine deltacoronavirus
Source: BMC Vet Res. 2024 Jan 10;20:22. doi: 10.1186/s12917-023-03863-2 (PMC10782762; doi:10.1186/s12917-023-03863-2)
Supplement: Supplementary file 1 — Supplementary Material 1: Table S1. Sequence information in this study [file 12917_2023_3863_MOESM1_ESM.docx]

Table S1. Sequence information in this study.

| No. | GenBank No. | Strain name | No. | GenBank No. | Strain name |
| --- | --- | --- | --- | --- | --- |
| 1 | MW816149.1 | CH7328 | 31 | MF642322.1 | CHN/GS/2016/1 |
| 2 | OM777140.1 | NTU/C253/21 | 32 | MG837133.1 | KNU16-07-P30 |
| 3 | MZ802777.1 | RBR-1/2016/Thailand | 33 | MG837132.1 | KNU16-07-P20 |
| 4 | MZ802776.1 | VUT-1/2016/Vietnam | 34 | MG837131.1 | KNU16-07-P10 |
| 5 | MZ802775.1 | NKP-1/2016/Thailand | 35 | MG837130.1 | KNU16-07-P5 |
| 6 | MZ802774.1 | CBR-3/2016/Thailand | 36 | MF280390.1 | CHN-GD-2016 |
| 7 | MZ802773.1 | CBR-2/2016/Thailand | 37 | KY513725.1 | CH/Jiangsu/2014 |
| 8 | MZ802772.1 | CBR-1/2016/Thailand | 38 | KY513724.1 | CH/Hunan/2014 |
| 9 | OK546242.1 | CZ2020 | 39 | KU665558.1 | CHN-LYG-2014 |
| 10 | MW854634.1 | 104-553 | 40 | KY926512.1 | KNU16-11 |
| 11 | MZ291567.1 | OH-FD22 P7 | 41 | MF431743.1 | SD |
| 12 | MT227371.1 | Peru/isolate/2019 | 42 | MF431742.1 | GD |
| 13 | MH118333.1 | P30 15 VN 1215 | 43 | KY364365.1 | KNU16-07 |
| 14 | MH118332.1 | P19 16 VN 0416 | 44 | KY354364.1 | DH2 |
| 15 | MH118331.1 | P1 16 VN 0116 | 45 | KY354363.1 | DH1 |
| 16 | MK625641.1 | CH/JXJGS01/2016 | 46 | KX834352.1 | Swine/Vietnam/Binh21/2015 |
| 17 | MK625640.1 | CH/JXJGS01/2016 | 47 | KX834351.1 | Swine/Vietnam/HaNoi6/2015 |
| 18 | MK625639.1 | CH/JXJGS01/2016 | 48 | KY065120.1 | CHN/Tianjin/2016 |
| 19 | MK625638.1 | CH/JXJGS01/2016 | 49 | KX118627.1 | P1 16 0115/PDCoV/2016/Lao |
| 20 | MW196362.1 | USA/IL/2014/026PDV P11 | 50 | KU984334.1 | TT 1115 |
| 21 | MH025764.1 | CH/JXJGS01/P50 | 51 | KU981062.1 | NH isolate passage 10 |
| 22 | MH025763.1 | CH/JXJGS01/P20 | 52 | KU981061.1 | NH isolate passage 5 |
| 23 | MH025762.1 | CH/JXJGS01/P7 | 53 | KU981060.1 | NH isolate passage 0 |
| 24 | MN025260.1 | Porcine deltacoronavirus | 54 | KU981059.1 | NH |
| 25 | KX998969.1 | P29 15 VN 1215 | 55 | KU051649.1 | Swine/Thailand/S5015L/2015 |
| 26 | MK993519.1 | CHN/Sichuan/2019 | 56 | KU051641.1 | Swine/Thailand/S5011/2015 |
| 27 | MG832584.1 | CHN-HN-1601 | 57 | KR265865.1 | Iowa459/2014 |
| 28 | MF642325.1 | CHN/QH/2017/1 | 58 | KR265864.1 | Minnesota292/2014 |
| 29 | MF642324.1 | CHN/GS/2017/1 | 59 | KR265863.1 | Ohio445/2014 |
| 30 | MF642323.1 | CHN/GS/2016/2 | 60 | KR265862.1 | Ohio444/2014 |

| No. | GenBank No. | Strain name | No. | GenBank No. | Strain name |
| --- | --- | --- | --- | --- | --- |
| 61 | KR265861.1 | Nebraska210/2014 | 91 | MZ388471.1 | CH/GX/PDCoV/1491B/2017 |
| 62 | KR265860.1 | Nebraska209/2014 | 92 | MZ388470.1 | CH/GX/PDCoV/1472A/2017 |
| 63 | KR265859.1 | Minnesota159/2014 | 93 | MZ388469.1 | CH/GX/PDCoV/1423/2016 |
| 64 | KR265858.1 | NorthCarolina452/2014 | 94 | ON494594.1 | CHN-SF-2018 |
| 65 | KR265857.1 | Illinois273/2014 | 95 | MH700629.1 | P20 16 VN 0416 |
| 66 | KR265856.1 | Illinois272/2014 | 96 | MH700628.1 | P12 14 VN 0814 |
| 67 | KR265855.1 | Minnesota455/2014 | 97 | MH700627.1 | NT1 0416/2016 |
| 68 | KR265854.1 | Minnesota454/2014 | 98 | OL900393.1 | CHN-SCMY2021-02 |
| 69 | KR265853.1 | Minnesota/2013 | 99 | OM256446.1 | PDCoV-CH-SDLY52-2021 |
| 70 | KR265852.1 | Illinois449/2014 | 100 | MZ772936.1 | BN |
| 71 | KR265851.1 | Indiana453/2014 | 101 | MN520209.1 | JS2019-A1414 |
| 72 | KR265850.1 | Michigan448/2014 | 102 | MN520208.1 | JS2018-YC15 |
| 73 | KR265849.1 | Michigan447/2014 | 103 | MN520207.1 | JS2018-QF49 |
| 74 | KR265848.1 | Minnesota214/2014 | 104 | MN520206.1 | HN2019-C132 |
| 75 | KR265847.1 | Minnesota442/2014 | 105 | MN520205.1 | HN2019-C115 |
| 76 | KR150443.1 | Arkansas61/2015 | 106 | MN520204.1 | HN2018-LH2 |
| 77 | KP757892.1 | CHN-JS-2014 | 107 | MN520203.1 | GX2018-1 |
| 78 | KP757891.1 | CHN-HB-2014 | 108 | MN520202.1 | GD2018-1 |
| 79 | KP757890.1 | CHN-AH-2004 | 109 | MN520201.1 | GD2018-186 |
| 80 | KT021234.1 | CH/SXD1/2015 | 110 | MN520200.1 | AH2018-94 |
| 81 | KT266822.1 | CH/Sichuan/S27/2012 | 111 | MN520199.1 | AH2018-93 |
| 82 | KR131621.1 | PDCoV/CHJXNI2/2015 | 112 | MN520198.1 | AH2019-H |
| 83 | KP981395.1 | USA/IL/2014/026PDV P11 | 113 | MN520197.1 | SD2018-4 |
| 84 | ON859973.1 | JSYC-2021 | 114 | MN520196.1 | SD2018-306 |
| 85 | ON402372.1 | HNZK-02-P60 | 115 | MN520195.1 | SD2018-304 |
| 86 | ON382562.1 | HNZK-02 P100 | 116 | MN520194.1 | SD2018-10 |
| 87 | ON968724.1 | CH/LNFX/2022 | 117 | MN520193.1 | AH2018-81 |
| 88 | MZ388474.1 | CH/GX/PDCoV/2081/2018 | 118 | MN520192.1 | ZJ2018-D |
| 89 | MZ388473.1 | CH/GX/PDCoV/1988A/2018 | 119 | MN520191.1 | SD2019-426 |
| 90 | MZ388472.1 | CH/GX/PDCoV/1539C/2017 | 120 | MN520190.1 | AH2018-322 |

| No. | GenBank No. | Strain name | No. | GenBank No. | Strain name |
| --- | --- | --- | --- | --- | --- |
| 121 | MT663769.1 | CHN-TS1-2019 | 153 | LC260044.1 | YMG/JPN/2014 |
| 122 | MZ802955.1 | CH-HLJ-20 | 154 | LC260043.1 | OKN/JPN/2014 |
| 123 | MW685624.1 | Haiti/Human/0329-4/2015 | 155 | LC260042.1 | MYZ/JPN/2014 |
| 124 | MW685623.1 | Haiti/Human/0256-1/2015 | 156 | LC260041.1 | IWT/JPN/2014 |
| 125 | MW685622.1 | Haiti/Human/0081-4/2014 | 157 | LC260040.1 | GNM-2/JPN/2014 |
| 126 | MT260150.1 | HNZK-04-P15 | 158 | LC260039.1 | GNM-1/JPN/2014 |
| 127 | MT260149.1 | HNZK-04-P5 | 159 | LC260038.1 | AKT/JPN/2014 |
| 128 | MN942260.1 | HeN/swine/2015 | 160 | MF041982.1 | SHJS/SL/2016 |
| 129 | MN781985.1 | CHzmd2019 | 161 | KY363868.1 | CHN-GD16-05 |
| 130 | MK359104.1 | CHN-GX01-2018 | 162 | KY363867.1 | CHN-GD16-03 |
| 131 | MN173782.1 | CHN-GX09-2018 | 163 | MF095123.1 | CHN-HG-2017 |
| 132 | MN173780.1 | CHN-GX12-2018 | 164 | KY293677.1 | CH/JXJGS01/2016 |
| 133 | MN173781.1 | CHN-GX81-2018 | 165 | LC260045.1 | HKD/JPN/2016 |
| 134 | MN173779.1 | CHN-GX11-2018 | 166 | KX022605.1 | USA/Nebraska145/2015 |
| 135 | MT263013.1 | CHN-HN-17 | 167 | KX022604.1 | USA/Nebraska137/2015 |
| 136 | KX361345.1 | 1215/PDCoV/2015/Thailand | 168 | KX022603.1 | USA/Minnesota140/2015 |
| 137 | KX361344.1 | 0313/PDCoV/0213/Thailand | 169 | KX022602.1 | USA/Iowa136/2015 |
| 138 | KX361343.1 | 0213/PDCoV/0213/Thailand | 170 | KT336560.1 | CHN-HN-2014 |
| 139 | MN249445.1 | CHN-JS-2017 | 171 | KJ567050.1 | 8734/USA-IA/2014 |
| 140 | MK330605.1 | CHN/Sichuan/2018 | 172 | KM820765.1 | KNU14-04 |
| 141 | MK330604.1 | CHN/Sichuan/2017 | 173 | KJ620016.1 | MI6148 |
| 142 | MK572803.1 | SCNC201705 | 174 | KJ584359.1 | NE3579 |
| 143 | MK355396.1 | CHN-SC2015 | 175 | KJ584358.1 | PA3148 |
| 144 | MK211169.1 | CHN/Sichuan/2017 | 176 | KJ584357.1 | KY4813 |
| 145 | MK005882.1 | Swine/CHN/SC/2018/1 | 177 | KJ584356.1 | SD3424 |
| 146 | KX443143.2 | CH-01 | 178 | KJ584355.1 | IL2768 |
| 147 | MH715491.1 | PDCoV/CHGD/2016 | 179 | KT381613.1 | OH11846 |
| 148 | MH708125.1 | HNZK-06 | 180 | KJ481931.1 | USA/Illinois121/2014 |
| 149 | MH708124.1 | HNZK-04 | 181 | JQ065042.2 | HKU15-44 |
| 150 | MH708123.1 | HNZK-02 | 182 | JQ065043.2 | HKU15-155 |
| 151 | KY293678.1 | CH/JXJGS02/2016 | **183** | **OP501870** | **CHN-HeN06-2022** |
| 152 | MG242062.1 | CHN-HeB1-2017 |  |  |  |
