# Supplementary material for: Phylogenetically evolutionary analysis provides insights into the genetic diversity and adaptive evolution of porcine deltacoronavirus
Source: BMC Vet Res. 2024 Jan 10;20:22. doi: 10.1186/s12917-023-03863-2 (PMC10782762; doi:10.1186/s12917-023-03863-2)
Supplement: Supplementary file 2 — Supplementary Material 2: Table S2. Primers used for genome sequencing [file 12917_2023_3863_MOESM2_ESM.docx]

**Table S2.** Primers used for genome sequencing

| **Primer name** | **Sequence 5’-3’** |
| --- | --- |
| P1-For | ACATGGGGACTAAAGATAAAAATTATAG |
| P1-Rev | GCAGCAGGCAGAGGCTTAGT |
| P2-For | GACGCCTATCTAACTCTCAGTAACG |
| P2-Rev | CTGCTTTGAATCCTGGAAGAGAG |
| P3-For | CATCCAAAGTTGTAGTACATGAAGC |
| P3-Rev | ATGGCAAGTTATTATCGAGTGTCAG |
| P4-For | GAGTCATTTATTGATGCTGAGTCTG |
| P4-Rev | TTGGTTGTATTGGGTCATATTCAC |
| N4-For | ATGCTGAGTCTGATGATCCACAAG |
| N4-Rev | AGCTACTGAAATAACACCTGTGTGG |
| P5-For | TAACCGTCGTGTTGGTGAGATG |
| P5-Rev | GGCGGAAAAACTTCATTACCTT |
| P6-For | CTTGTCATGGGCACGCTAATAG |
| P6-Rev | TTCCCACGCATAACAAGTGTAGAT |
| P7-For | CCCTCAATGCAGAAGACCAGTC |
| P7-Rev | TGTTGATGTAAAGATGTGAATACCAGAT |
| P8-For | CTTTCAAGATACCAGAGCAGATAATG |
| P8-Rev | TGGAGTGGCTGTGTTGTTGG |
| P9-For | CTGGCTTAAGAATGTTGTCTACTGC |
| P9-Rev | GCAACATAACCATATTGAAACCACT |
| P10-For | CTAACACTTTTTGGATACCGAACAC |
| P10-Rev | ACTCAAACAGAGCTTTTACCCTATC |
| P11-For | CGGAATGGGAGCGTGAGG |
| P11-Rev | CGTCACTAGAACCCGTTACTCG |
| N11-For | TTGCTGATGCTGCTATGAAGTCTAT |
| N11-Rev | GACTAGATCCACAGGTGCAGTCAT |
| P12-For | CGTGCTCATATACCACATCCTG |
| P12-Rev | ATTGCCAAATGCCGTATTAGGT |
| P13-For | TAAAGTGTGAGTGTATGGATAGTGATG |
| P13-Rev | GGTTGTTACCAGAGACTACCACTTC |
| N13-For | ATGGCAACCCACTTGAGTATGAT |
| N13-Rev | CTGATAGTACTTGGCAACATTCATTAG |
| P14-For | TTACCCTAAGTGTGATCGTTCTATG |
| P14-Rev | AGATACCAAAAACAGAACCATTAGC |
| P15-For | CTACCAGCATGTTACGCAGACTAC |
| P15-Rev | CGCTCCTGCATGTACCATTATATC |
| P16-For | CACAACTGCACAGTATATCTTCTCG |
| P16-Rev | AAGGTGACGAAATTGTTCTCCAG |
| P17-For | AGCACACGCCATAAAACCTAAC |
| P17-Rev | TTGCGTGGTATGTAAACATTAAATC |
| P18-For | GCTACACATAAACTCAACTTGGCTC |
| P18-Rev | GCATGAATCTAATTTGTTGGTTGTC |
| P19-For | TCATTGGCGGAACTCACACAC |
| P19-Rev | TAGAGACTGGTGGAATTACTCGTGG |
| N19-For | ACATCAGCTTGTCAACCATGTTTAC |
| N19-Rev | GTTTATGTAAGAAGCGATGTGCAC |
| P20-For | CGGCTCGTGAGTTAGAGAAGAAG |
| P20-Rev | TGACATGCCATTATTTACTGCTGAC |
| P21-For | CCAGACTTGACAGCGTTACCATC |
| P21-Rev | GGGAACAAACTAAGTCAGCGATG |
| N21-For | TTACCATCAATGGTAACACATCCTAT |
| N21-Rev | AGGCTTTGTAGTCCTGATCAACAG |
| P22-For | ATATTACCAACTTCAAGGGTGACTAC |
| P22-Rev | GTTATAGATACCAACATCCCACTGAG |
| P23-For | GCAAGCTGATTTCATACAATTAACTG |
| P23-Rev | TTAACGACTGGTGTGAGTATTGGAG |
| P24-For | ACAGATCATCTAAGAAGGACGCAG |
| P24-Rev | CCACGCTCCTGAGGTCTTCC |
| N24-For | AGAAGGACGCAGTTTTCATTGTG |
| N24-Rev | GTTGAAGGGGTCAACTCTGAAAC |
| P25-For | CTCCTAATGATACCCCAGCAAC |
| P25-Rev | TGCTCCATCCCCCCTATAAG |
