# Supplementary material for: Phylogenetically evolutionary analysis provides insights into the genetic diversity and adaptive evolution of porcine deltacoronavirus
Source: BMC Vet Res. 2024 Jan 10;20:22. doi: 10.1186/s12917-023-03863-2 (PMC10782762; doi:10.1186/s12917-023-03863-2)
Supplement: Supplementary file 3 — Supplementary Material 3: Supplementary file 1. The genome sequence of PDCoV strain CHN-HeN06-2022 [file 12917_2023_3863_MOESM3_ESM.docx]

**Supplementary file 1. The genome sequence of PDCoV strain, CHN-HeN06-2022.**

ACATGGGGACTAAAGATAAAAATTATAGCATTAGTCTATAATTTTATCTCCCTAGCTTCGCTAGTTCTCTACCGACACCAATCCAGGTGCGTCTGCCACCAAGTTGGCTACCCTTCTCAGGGGCGCTTTTGCGCTTGCTCACCATTAGATTACCTGGAAACCAGCCATTCAGGTTGGAGTTTCCCCAGGCTCTTTTGTGTGGGCATTAGCGGCTTGTGGTTTTTGCACAAAATCTAAGCTACTTACCGTTCCTCTGACCATCCACCACTTCTATAGACAGCACTGACTACCGTAGGGTTTAAGTCACACCGGTCTGCACCGCCCGTCAGCGGACACATTACCCAGCATAGCACTCCTTGCACCGAGCCTAGGTAGGATAAAACCCCCTACCGGGTGACTCTTAAGGCGTTTCCTCCACGGGATAGCCACTAGTCACTAGGTGTAAGTGATCTGATCTGGGCGTATTGTGTTGCGCAAGTGTGATACCCATAGGAGCGTGGAATCCTATTCTGCGGCTCAGTGCCTGATATAGCTGTGAAATGGCCAAGAACAAGTCCAAGCGCGACGCTATCGCGTTGCCTGAAAATGTACCACCACCTCTGCAACTTTTCATCCACGTTGCAGCTGCTGAAGAGGGTCACCCTAAGGTTACTACTTACCTTGGCAACTATAACCTCTACGCTACCAAGGCTCCGCCTGGCGTGCAGGTTCTTAGTGCTAAAACCTCTCTTACTGATTTTGAGAATGTCTTTGGAGCTCAACCCACCTTGCGATCAATTCGTAATCTGGTGTGTGAGGCTCGCATGGCTGAATGGACAACTTCCAAGAATGCCTTTGCACTCAAAGCCACTCAACTTGACTACTCTGATGCCGTTTTGAGGGCAATGATTCGTTTTTGCCCTCCAAAGGTGTCCACACTTGCTGCCTTTGCTCTTTTTGGCCGATTGGTTAAAATTGAGGACAAGGAACTTGCTGAGTTAGCTCGTGATACTGCCCTTGAGCTGGCGTACACTGCTAAAATTGGTACATCTCTTGCTGACACGAGATCTGTCTCACTTATTCATAAGGACGCCTATCTAACTCTCAGTAACGAGGTTGTTGGCGTAACTTTTACTGCCGCACTTATGGCAAAGGCTACCACTGTTAATGGAGCAATGCAATACTCAAACTTTTACCTTTACCCTCGCGCCACTATCAAGGTGACCGATGGTAAGGCTGAAGCAATTGCAACTAAGCCTCTGCCTGCTGCCACTAAAGGCAAGCCAATCACAGAGGATGTCAACCTCCTCCCTGACTATCAGCAGCTGCTTGTTGATCAAGTGACTGGCACTGAGGTTAAGGTTGGAGCCTTAACTTATGTTAAGACCACTGACCTACCACCCCTTTACTTTCCTAAAGTTAAAGGCGGTGTTGTTGGTATTGCACTTAAGCAGCAGGGCAATGTGGCTAAGAAGCTCAATGTAGTCTTCCATGCTCAACCTGATGATGTTCTGCTAGCTTTCATACAACTTCAGCAATTCTTGAACCGCACTTCGGATTCAAGTGTTGAAATTACTGATAGCCAGAGTTATGAAGTCTCTCCAACTGTGACGGTAAAAATAGGTCCGTCTAAACCTGGAGATGTCATCGTGGCTACTGATGAGGAATACCTTAAATGCTTTGAAACCCCCGAGGTAGGTAGGCTCTATAAGGTTTTCCAAACTCAATCTTGGGCTATCATTGAGCGTTCCTTTTCCAGGATCCGCGTGTCCAAAGCTTTATCAGCATTTATAAGTTTTCTGCAAAACCTTGCAGATAACTTTACTGCACTAAGTGGTGTTGTCACTGCACTCATTCGTGAACTCCAGGATCTCACCCTGGATGTGGCGACACGTATCACTAACATACAATTTGTTTACCGCGCTGGTAAGCTTATTGTCGACACGACAAGTGTCATAGCTAAACTTTTCCAGCCATTTTGTGATTTTATATCACCTTTCCTTCGGAAAGTTGCTGGTTTTGCAATTTACACTGTTGGTAACCGCATGCTTATGTTTACCAGCACTGGCACCTTTCTTCTCACAAAGGCAACTACTAAGATACTCAATAAGGCAAAGTACATCTTTGATGTTGAGCCTGAGTATCCAGTAGATGTAACAACATCCAAAGTTGTAGTACATGAAGCACTCCAGCAAATCGACACTAAGCCTGCTAGAGCTCTAGAGGCTGTTGATGTTGTTGTTGGTAACACTGTACTGCAAATGGCTACTGATGGCACTGCGTTCTACCCATCAGATGGTACGCATGCCTCTTTACCAGGATTCAAAGCAGGTTCGGATGAGCTCCTCATAAGCTTCAACTGTGACCTCTTTGATGATGAGACTAATGCTCAAATCAACGAAACACTCGCTGCATATGAGCTTAACCAACTAGTAGCTCCAGGTGATTCTACACCGCGTCAAATTGCGACGTTGGTTGTCGATACACTTGCAGATGCTATAACAGACCACTTTCCGGAGAAAACCATTGATCTACCTGAAGACTATCAAGTCTTTTCTGATCATGATGACCTCCCACTTGCACAATACCACATCCCTGATCACCTGAGCCTGTATATTCAGGCTATGGAAGGTGAAGATGATAGTGGTGATGAAATATGTATTGAGGACGATGATTACGACTGTCCTCAAGCCGACGAAGACACAGAAGGAGTAATTCCCCAACAGTGGGAACTTCCTGATGTTGATAAATTTTTACTCAAGATCCAGGAACGGAAGACCAGCAGCGACGAAGTACTTAGCGTCGACGTCTATCCTAAACCAGATCCGGTTGGCAATGTTGGGGTTGACGACAGCGCGTCGGAAAAGAAGCCAAATGGAGACCCAGTACCGGATCCTGAGGTTCATCCAACACTAGAGAGTGTGGATGTTGAACAACCAACCGAAACAGCAAACCAGGCTGTTGAAGACAAACCTTCTGATACCACCTTTGTGGTTGATGAGGAACAATTACAAGAATCAACACCAGAACAAGAGCTCCGCTCCTATGAAGGGGAGTTTGATTCTGATGATGAAATTATTATTCCTATAGTACCAGTAACACCTGCGGATTTAAAACCACAGACTATTACTATAAAGGAGTACTTTAAGTCTGAAAAACTTGAGACTATTAACGAAGGATCTACAGAGTCAGTTGCACAGTCTGATGATTCGTTTGACGAGTCATTTGTTGATGCTGAGTCCGATGATCCACAAGACCTTGCTGTATATGATGATACAACAATTATAACGGACAGCACTGATGTAGGCGATGAGCCTGAGACAACTCTAGCTACCATCGTTAACACACCTCTGACACTCGATAATAACTTGCCACCTGAAGCCATTAAACAACCCAGCCCGACTAAGGTTGAGTTAGTTGTTGGTGAATTGGCAAGTATTAAATTTGACAATTCTGTCCTAGTCAACCCTGCTAATGCGCAATTAACAAATGGCGGTGGAGCTGCCCGTGCAATTGCAAAATTAGCTGGTCCAAAATACCAAGAGTACTGTAATAGTGTGGCTCCTATCTCAGGACCGCTTACCACGGACTCTTTTGATGCCAAGAAACTTGGTGTAGCCTGCATCTTGCATGTAGTGCCACCCAAAGGTTCTGACCCTAATGTACAAGAACTCCTGTATCAAGCTTACAAGAGTATCCTTACTGAACCAGCACACTATGTTATACCTATACTAGGTGCTGGTATCTTTGGATGCAACCCAGTCCACTCTCTGGATGCGTTCAGGAAAGCATGTCCAAGTGACATAGGTCGTGTCACCCTTGTCACTATGAACAAAAACCATTTGCAGGTGTGGGATGCTCTCAATAGGACCATTGTACGCACCACTACAGACTATGATCAAGTTACCACCAAGGCCCTTACACCCCAGGGAGTGTTAGAAGCCAATCTCTTTGATGGTGAGGACTTTGTTCAAGAACCAAAACCCGGTCAAATCTACCTTGAGGTTACTGAAGAAGTTCAGAACCAAGCCAAGGAACTTGACCTTAACCTTCAGCAATACTGCGTCTACCTGAAGACTTGCCACCATAAATGGGTTGTGAGTCGTACGAACGGGTTGATGCATCTAAAACAAAAAGATAACAATTGTTTTGTTAGTGCAGGTGTAAACCTGTTTCAAAACACTGCTTATCAACTTAGACCTGCTATTGATGCTCTCTATAGGGAGTATCTCAATGGTAATCCAAACAGATTTGTTGCTTGGATCTACGCATCCACTAACCGTCGTGTTGGTGAGATGGGTTGTCCACAGCAAGTTATTTCTTTGCTCGTTAGTAACTCTGACGCAGCATTTTCAGCAACTACAGCCTGTTGTAACACCTACTTTAACCACACAGGTGTTATTTCAGTAGCTCGTGAATATGACCCAATACAACCAAAGGTCTACTGCATGAAGTGTGATGTGTGGACTCCCTTTACACCCCAGAGTGGAAAAGGTGCAGTTGCAATTGGTACTTCTGCAGATGAACCTACCGGTCCTGCCATTAAATTTGCCGCAGCTCACTGCTGGTACACTAATGGCAAGAAAACAGTTAATGGTTATGACACTAAAGCTAATGTTGTAGCTACTTATCATAGGTTTGACGTGCCTAAGCCTCAACTTGTTGAGGACGTGGTCGCGCTGCCTACTAAAAATGACTTTGAAGTTCTCAATGTTGAAGAACTGCCACAGGATAGTGTGCTCCATTTGGACCCACCTCCTGTACAGGCCTTACAACCTAAGGCTAACCAACACATTGAGATTTTAGAAAACCCAGATTATCTGGACATTTTGGATCTTTGGATTCGTAAACCCAAATTCATCCTCGTAAAGTCGTGGAGTGTTTTGGGTAGAGCACTATGTAAGGCAGGTAAAGTTGTCTTTGTCAGTGCTTCGCTTTTGACCCGTTTCTACAATTACCTTGTAGAGATTGGTGCTCTTGACTCAACAATAAGGTTGTCAGTCGATCTTACCTGTAAATTTGTTAGAACGGTACTCCCATCGTCTAACACTGTACACAAAACTTGTCTTGGTCTGTATTATTCAGCCCAGACACTTTTTGTTTCTTTAGCACCATTCCTTATGTTACCAGCTGTAGTTAGTCTGCTTAATTCAGGCTATACAATTGGCACATATTTGTATGCAAAAACTGGCTGGCCTTGTAATTACAATGCCACGCAACACTTTGATTATAATTCTTACTGTGCAGGTGACTTGGTTTGTCAAGCCTGTTTTGACGGTCAAGACTCCCTACATTTGTATCCGCATTTACGTGTTAATCAGCAGCCCCTTCAGACCACTGACTACACTGTTTATGCGCTTTCACTAATACTACTATTAGCTAACATGACTCTTGTCATGGGCACGCTAATAGTTACTTTCTTTGTGAACTTCTATGGTGTGCAAATACCATTTTATGGTACACTTCTGATAGATTATCAGTCCGCGCTGATGATGACTTTCTCAGTGTACTACTTTTATAAGGTAATGAAGTTTTTCCGTCATCTCACACATGGATGTAAAATTCCAATGTGTATGGTATGTGCTAAACTTCGTACCCCACCTACTATAACAGTTGAGACTGTCGTTCAAGGCAGGAAATACCCATCTGTTATTGAAACAAATGGCGGGTTTACAATTTGTAAAGAACACAACTTCTATTGCAAAGACTGCTCTCTACAAACACCCGGCACTTTCATCCCGACAGAAGCTATTGAGTCGCTCTCACGAGCTACCAGGCTTAGTGTCAAACCAACAGCACCAGCATTCTTACTTGCTAGAGATGTTGAGTGCCAAACTGATGTTGTCGTTGCTCGTGCAATGCATAACCAAAATGCGCATGTGTGCATTTCAAAATACTCAGATATCCGTACCGTTGACCAACTACTTAAGCCTACTCCACTGTTTTCATACACTCCCGATGTTATCATCGCGGCAGACTTTGACAACAGAGGTAGTCTTAAGACAGCTAAAGAATTAGCTGTGGTTTTGTCAATGGACCTTAAACGTACTATAATTATCATTGATCAGGCCTATTCTAGACCCATTGATAATTATCAGGAAGTTGCTTCTCGTATTGAGAAGTATTACCCAGTTGCAAAGATCACACCCACAGGTGACATCTTTACAGACATTAAGCAAGCGACCAATGGCCAAGCTAGTGACTCTGCTATTAATGCAGCTGTTCTGGCTGTCCAACGTGGTCTTGATTTTACAATTGACAACCCTAACAACATATTGCCACATTACGCCTTTGACTTTTCAACCCTTACTGCAGAAGACCAGTCTACCATTTTGGAGAGTGGTTGTGCTAAAGGCAATCTCAAAGGCACTAATGTTGGTGTTGTTCTTTCAGCTAGCCTTGTTACACGTCTTAGTCAGCAGGCTATACGTGTGATCGCTAATGCTGCTTCACGTAATGGTGTTACATGCGCTGTTACTCCATCTACACTTGTTATGCGTGGGAATATTGCAACTCAGCCTTTGACTCGCATCAAAGCTGGTGCACCTCCCATGCGTCAAAAAATCTTATGTGTTATCCTGGCACTTGCTATTGTGTACTTTGCTGCTATGGCTTTTGGCTTTTTGGCAAGTCAAATTACGCTTAATACAGTGCCTACGATTAAATCTGATATCCGCGCCTCTACCTTCTACGTTGTTAGAGATGGAGTCCTGGACACTGTTCGTTCAAATGACAAGTGCTTTGCAAATAAGTTTTTGGCATTTGATAGCTTCATCCAAGCACCTTACACTAATTCACCTGACTGTCCAGTTGTTGTGGGAGTTGTTGATGTAACGACGCACTCTATTCCTGGAATTCCAGCAGGTGTCATTCATAGAGATGGTCTCATACTTAACATTTATGAACAGTCTCTTTATGAAACCCAACAGCGTCAGTCTATGGTTAGGGATGCGTTGTCACTTAAGACAGCAAACCTCTTTAACCTAGGCAAGCGCGTTGTAGTAGGATACACTCAACATGAAGTTGTTGTGGGTACCTCCTATTTTAATTCTCCTGCACTTTTTAACGCAAAGTGTACCTTCTTACAGTACCAGGACACTAGACAACTCTATTGCTATGATACTGTTCCTACTGAACATAAGCTTTACTCTGATGTGCTTCCGCACGTCGAGTATAAGGCTATTGACATTAATGGTGATCTTGTTCCTTTCAAGATACCGGAGCAGATAATGTTCTATCCACATATTGTGCGCTATACTAGCAATTCCTATTGCCGTATGGGGCATTGTTTTAATACTAACCCTGGTATTTGCATTTCATTTACGGACGAATTTCCGTATAGTGAAAATGTCAAACCTGGTGTGTACTGTGCTGATACCTCTTTGCAGTTGTTTTCAAACCTCGTTTTGGGCACTGTATCTGGTATTCACATCTTTACATCAACAGCTGCATTGCTTGGATCTACTATTGTTATCATACTATGCGTTGTTGCTGTTCTTGCAGTTCAGCGATTCTTCAAGGAGTACACAACTTTTGTTATGTACACTTGTGGTCTTGCTCTTGTCAACATTGTGGGCATTGCACTTATGTACAAGTGCCTTGTCTTCGCGATCTTCTATTATGCAATCTACCTTTACTTTGTCCTTACCTTCCCCTCCTTTAAGAGGAATGTGGCATTGTTTTACTTCGCTGTAGTGATCGTGCCGCACGTGAGTAACATGCAATTGCTTGCGCTCATTGTGTGTAGCATTATCTACTTTCTCTACACCTATGTTCATACTGTAGCTAAGACAGCTGGGAAATTCTCTTCCTTCTTAGACGCAGCTAAAGCTACTTTTGTCATTGACAATGAAAAGTACGTGTTGCTTAAAGACCTCGCTGGTGCTGAATTTGACCAGTATCTGGCCTCTTACAACAAGTACAAATATTTTTCTGGTACTGCTTCTGATAAGGATTATGATAAGGTCTGTATGGCATTTCTTGCCAAGGCCTTGTCATCTTTTCGTGAAGGAGGCGGTTCACAGTTGTACACACCACCTAAATTTGCAGTTGTTCAGAGTCTTAAGACCAAGCTGCAAGCAGGTATCAAAATCCTCCTGCACCCTTCAGGTGTAGTTGAGCGATGTATGGTCTCAGTTGTCTACAATGGATCTGCATTGAATGGCATCTGGCTTAAGAATGTTGTCTACTGCCCACGCCATGTAATTGGAAAATTCCGTGGTGACCAGTGGACTCACATGGTCTCAATTGCTGATTGCCGCGACTTTATAGTCAAGTGTCCAACACAGGGTATTCAGCTAAATGTCCAGTCAGTTAAGATGGTAGGAGCTCTCCTCCAGTTAACTGTTCATACCAACAACACAGCCACTCCAGACTATAAGTTTGAAAGGCTCCAACCAGGATCGTCGATGACAATTGCTTGTGCTTATGATGGCATTGTACGGCATGTCTATCACGTGGTCCTCCAACTTAATAATCTTATTTATGCAAGCTTCCTTAACGGAGCTTGTGGTAGTGTGGGTTACACTCTTAAGGGTAAAACACTCTACTTACATTACATGCACCACATTGAGTTTAACAACAAAACTCATAGTGGTACAGATCTTGAAGGTAACTTCTATGGCCCCTATGTGGATGAGGAAGTTATTCAGCAACAAACAGCATTCCAGTATTACACTGATAATGTTGTTGCTCAATTATATGCACACTTACTGACTGTTGATGCTAGACCAAAATGGCTGGCACAATCTCAGATAAGTATCGAGGATTTTAACTCATGGGCTGCTAACAATTCCTTTGCTAACTTCCCATGTGAACAAACTAATATGTCCTACATTATGGGACTCTCGCAAACTGCTCGAGTCCCTGTAGAACGTATCCTCAATACCATTATACAGCTAACCACCAATAGAGATGGTGCTTGTATTATGGGATCTTATGATTTCGAGTGTGATTGGACGCCAGAGATGGTATATAATCAGGCTCCAATTTCATTGCAGTCAGGAGTAGTTAAGAAAACTTGTACGTGGTTCTTCCACTTCTTGTTTATGGCTATTACCATGCTACTCGCTGCCATGCATGTTTTCCCTGTACACTTGTACCCAATAGTACTGCCATGCTTCACTGTTGTGGCATTCCTGTTGACTTTAACCATTAAACACACTGTTGTGTTTACTACTACATATTTGCTTCCGTCACTTTTGATGATGGTTGTAAATGCTAACACTTTTTGGATACCGAACACATTTCTGCGCACTTGCTACGAAACTATATTCGGTTCCCCAATTGCTCAGCGACTGTATGGTTACACTGTTGCTCTTTATATGCTGATCTATGCTGGACTTGCAATCAACTATACGTTGAAAACACTCCGGTATAGAGCAACTTCATTCTTATCTTTTTGCATGCAGTGGTTTCAATATGGTTATGTTGCACACATTGCGTACAAACTGCTTAATAAACCCTGGACAGAATCACTACTCTTCACAGCCTTCACAATGCTAACCAGTCATCCTTTGTTGGCTGCTCTTAGCTGGTGGCTAGCTGGTCGCGTAACTCTGCCCATTATCATGCCTGACTTAGCTATTCGTGTTTTGACGTATAACGTCATTGGCTATGTCATATGTGTTCGATTTGGCCTTATGTGGCTTGCAAATCGGTTCACAACTGTACCTATGGGCACATACCAGTATATGGTGTCTGTAGAGCAACTTAAGTACATGATGGCAGTTAAGATGTCCCCACCGCGTAATGCGTTTGAGGTGCTTATAGCCAACGTTAGACTTCTTGGTTTGGGTGGAAACCGTAACATTGCTGTTTCTACTGTCCAAAACAAAATTCTTGATGCAAAAGCTACTGCTGTTGTTGTTGCTAACCTTCTTGAAAAGGCTGGCGTCACAAACAAGCACGCTATTTGCAAAAAGATTGTGAAACTCCACAATGATACCCTTAAAGCCACCACTTATGAGGAGGTTGAGGTAGCACTTGTGAAACTTCTTTCTCACATAATTGAGTTCTTGCCAACTGATCAGGTAGATGCTTATCTAGCTGATGCGGCCAATGCTCAACATGTTAATACCTATTTCGATAACTTGCTTGAGAACAAAGCTGTTGTTCAGGCTGTTGCCGATATCAACATTAATCTGGATTCTTATAGAATTTATAAGGAGGCAGATGCTATTTACAAACGATCTGTTGAGATGAACGAATCTCCACAGGAGCAAAAGAAAAAGCTTAAAGCTGTCAACATTGCAAAGGCGGAATGGGAGCGTGAGGCTGCTTCTCAGCGTAAGCTTGAAAAGCTTGCTGATGCTGCTATGAAGTCTATGTATCTTGCAGAACGTGCTGAGGATCGTCGCATTAAGCTAACCTCTGGACTTACTGCAATGCTTTACCATATGCTTAGACGTCTTGACTCAGATAGGGTAAAAGCTCTGTTTGAGTGCGCTAAGGCACAAATCTTGCCAATACATGCTGTAGTTGGAATTTCTAATGACAACCTTAAAGTTATTTTTAACGATAAGGATAGCTACTCTCATTATGTAGATGGCAACACACTTATACATAAGGGAGTTCGCTACACTATTGTGAAGAAACTCTCCTTAGATAATGCACCTATTGAAGGCGTACCAGAAGAATTCCCTGTGGTCGTTGAGACTGTTAGGGAAGGTGTGCCCCAGTTGCAAAACAATGAGCTATGTTTGCGCAATGTTTTCACTGCTCAGAACACAGCTCAGGACTTCAATGGCAATGAATCCACTGTAAAATCTTTTTATGTTACTAGAACCGGTAAGAAGATTTTGGTTGCCATTACATCAACTAAAGACAATCTTAAGACTGTGACCTGCCTTACTGAGACCGGTAAGACAGTCCTTAATTTGGACCCTCCTATGCGCTTCGCACATACCGTAGGTGGAAAACAGTCTGTTGTCTATCTCTATTTTATTCAGAATATTAGTTCACTCAACAGAGGTATGGTTATTGGCCACATCTCTGAAACTACTATCCTTCAGGCAAGTGGCACTCAAATTGAGTACCAGCAAAATGCCTCTCTTTTGACCTATTTGGCTTTCGCTGTAGACCCTAAGACAGCCTACCTTAAGCATCTTGCTGATGGTGGGTCTCCTATACAGGGTTGTATTCAGATGATTGCTACTATGGGTCCTGGATTTGCAGTTACTACTAAACCACAACCTAATGAGCATCAGTATTCTTATGGTGGTGCTTCAATTTGTCTTTATTGCCGTGCTCATATACCACATCCTGGTGTTGATGGACGGTGCCCCTACAAAGGCCGCTTTGTTCACATCGACAAAGATAAGGAACCTGTTTCCTTCGCCTTGACTCATGAGCCATGCAGTTCTTGTCAACGGTGGGTCAATTATGACTGCACCTGTGGATCTAGTCTGCAGAATTCGGCTTATTTAAACGAGTAACGGGTTCTAGTGACGCCCGGCTAGAACCCCTGCAGCCTGGAACTCAACCAGATGCTGTAAAAAGGGCCTTCCATGTGCATAATGATACCACCTCTGGTATATTCTTAAGCACAAAATCTAACTGCGCTCGGTTTAAAACCACACGCAGTGCCTTGCCTTTACCTAACAAGGGAGAGGTTGAATTGTACTTTGTTACTAAGCAGTGTGCAGCTAAAGTCTTCGAAATCGAGGAGGAATGCTACAACGCTCTTAGTACAGAGCTTTATACTACTGATGATACATTTGGTGTCCTTGCCAAAACTGAGTTTTTCAAGTTTGACAAGATACCTAATGTCAATCGTCAGTATCTGACTAAATATACACTCCTGGACTTGGCTTATGCTTTACGTCATTTGTCAACATCTAAGGACGTTATTCAAGAAATTTTGATCACCATGTGCGGAACCCCTGAAGATTGGTTTGGGGAAAATTGGTTTGATCCAATTGAGAACCCATCCTTTTACAAGGAGTTCCATAAACTTGGAGATATTCTTAACCGTTGTGTTCTTAATGCCAATAAGTTTGCTAGTGCCTGTATAGACGCTGGTCTTGTTGGCATATTAACACCCGACAATCAAGACCTCCTGGGTCAGATCTATGACTTTGGAGATTTTATTATTACACAACCAGGTAATGGATGTGTAGACTTAGCATCCTATTATTCTTATTTAATGCCCATTATGTCCATGACTCACATGTTAAAGTGTGAGTGTATGGATAGTGATGGCAACCCACTTGAGTATGATGGATTTCAGTATGACTTCACGGACTTCAAGCTTGGCTTGTTCGAGAAGTATTTTAAGTACTGGGACCGTCCTTACCATCCTAACACTGTTGAATGTCCAGATGACCGTTGCGTATTGCACTGTGCGAACTTCAATGTGTTGTTTGCTATGTGTATACCTAATACGGCATTTGGCAATCTTTGTTCAAGAGCTACTGTTGATGGCCACCTTGTGGTCCAGACAGTGGGTGTACACTTGAAAGAACTTGGTATAGTCCTTAACCAGGACGTTACCACACATATGGCAAATATTAATCTAAACACTTTATTGCGATTGGTAGGTGATCCCACCACCATTGCAAGTGTCTCAGACAAGTGTGTAGATTTAAGAACTCCTTGTCAGACCTTGGCTACTATGTCTAGCGGAATTGCTAAACAGTCAGTCAAGCCCGGGCATTTTAATCAACACTTCTACAAGCATTTGCTTGATAGTAATCTATTAGACCAACTTGGAATAGACATTCGCCACTTCTACTATATGCAGGATGGTGAAGCGGCTATCACAGACTACAGCTACTACAGGTATAATACCCCCACGATGTTAGATATCAAGATGTTCTTATTTTGCCTTGAGGTGGCAGATAAGTATCTTGAGCCCTACGAAGGTGGATGTATTAATGCACAGTCAGTTGTGGTCTCTAATTTGGACAAATCAGCGGGCTACCCCTTTAACAAGCTGGGTAAGGCTCGTAACTATTACGACATGACTCATGCCGAGCAAAATCAACTGTTTGAGTATACAAAACGCAATGTTTTGCCTACACTCACTCAGATGAACCTTAAGTATGCAATTTCAGCCAAGGATCGTGCTCGCACTGTGGCAGGAGTGTCTATAATTAGCACCATGACTAACAGGCAGTACCATCAAAAGATGCTGAAATCTATTTCACTTGCACGCAATCAGACCATCGTGATTGGAACAACCAAATTCTATGGTGGTTGGGACAACATGTTACGACGACTGATGTGTAATATCAATAATCCCATTTTAGTGGGTTGGGATTACCCTAAGTGTGATCGTTCTATGCCAAACATGCTGCGCATTGCCGCTTCGTGCTTGCTAGCACGAAAACACACTTGCTGTAATCAAAGCCAGCGATTCTACCGTTTGGCTAATGAATGTTGCCAAGTACTATCTGAAGTGGTAGTCTCTGGTAACAACCTCTATGTAAAACCAGGTGGCACTAGCAGTGGTGATGCAACCACAGCTTATGCCAACTCGGTATTTAACATCTTACAGGTGGTTTCTGCTAATGTAGCCACCTTCTTATCAACTTCCACCACGACACATCTTAATAAGGACATCGCGGACTTGCATCGTAGTCTTTATGAAGATATCTATCGTGGTGACTCTAATGATATCACCGTCATCAATAGATTCTACCAGCATCTCCAAAGTTACTTTGGACTTATGATATTGTCTGATGATGGTGTCGCATGCATAGACTCAGCCGTTGCAAAGGCTGGAGCTGTTGCTGATCTTGATGGTTTCCGAGACATTTTGTTTTACCAAAACAATGTTTACATGGCAGACTCAAAGTGTTGGACAGAAACTGACATGAATGTTGGCCCTCATGAATTTTGCTCACAGCATACTGTGTTAGCAGAGCATGATGGTAAACCTTACTACTTACCTTACCCAGATGTCTCTCGCATTCTGGGTGCATGCATCTTTGTGGATGACGTTAATAAGGCTGACCCTGTTCAGAACCTTGAACGTTACATCTCACTTGCAATTGATGCATATCCTCTCACCAAGGTTGACCCTATTAAGGGTAAAGTCTTCTACTTGTTACTAGACTACATACGTGTTCTTGCTCAGGAGTTACAGGATGGTATCCTTGATGCTTTCCAATCACTCACTGACATGTCGTATGTAAACAACTTTATGAATGAGGCCTTTTATGCTCAGATGTATGAGCAAAGTCCTACACTACAGGCCAGCGGTGTTTGTGTGGTGTGTAATTCACCCACTATACTGCGCTGTGGTGATTGCATTCGTCGACCACTACTTTGTTGCGTCTGTGCCTACCAGCATGTTACGCAGACTACACATAAACGTATCATTGCTATCAACAACTACATTTGTAGTGTTGAGAATTGCAATGAGGACAATGTTGAAAAACTTTTCATTTCTGGCACTGCGATCTATTGTGAGAATCACAAACCCACGCTGTGCATACCCATTGTAGCTAATGGTTCTGTTTTTGGTATCTATCGCCACACTGCCCGTGGTAGTGATGACATAGACCTCTTTAACGAGCTTGCTACATCTAACTATGACACTATTGAACCTTATCAGAAGGCCAATCGTGCACCTTTATCACTTATGCTCTTCGCTGCTGAGACCATTAAGGCACTCGAGGAGTCTATCAAGAAGTCATACGCTACCGCAACTGTCAAGGATGTGTATGACCAACGCTTCATTAAACTTCTATGGGAACAGGGTAAAAAGCCGCCACCCATAACGAAGAACCACATTTTCACTGGCTACCATTTTAACAAGAATGGAAAAACCCAAGTTGGTGATTACATTCTTGCTAAAACAGATGGCAGTGACACTTATACTTACAGAGGAACATCTACCTACAAACTTCAAACAGGTGATGTTCTAGTCTTAATGGCACATGTTGTTACACCGCTCTCAGCACCCCCTGTGCTAACGCAGACAACATATGTCAGAAAATCACTTTTACCCGACTCTGTTGGTGCGTCTTATTATGTGCAACATTTCAAGTCATATAATGAGATAGCTATGCAGAGGGTTACAACAGTATTAGGTCCACCAGGCACAGGTAAGTCAACCTTTGCTATTGGTTTGGCTAAGTACTTTCCTAGTGCACGTATTTGCTACACTGCGTCTTCGCATGCAGCAATCGATGCACTCTGTGAAAAAGCTTTCAAGACAATACCTGTAGGCCAATGCAGTCGTATCGTACCCACACGTACAACTGTTGAGTGCTTTCAGGAGTTTGTCGTAAATAACACAACTGCACAGTATATCTTCTCGACTATCAATGCCTTACCTGACATTAAGTGTGACATTGTAGTCGTAGATGAGGTTTCTATGTTGACCAATTATGAGCTTTCCTCTGTGAATGCTCGTTTGGTTTACAATCACATTGTGTATGTTGGTGATCCTTATCAGTTACCTTCACCTAGAACTATGCTTACGTCTGGCCAGCTTTCGCCAGCTGACTATAACGTAGTTACTGATATAATGGTACATGCAGGAGCGGATGTTATGCTCGACATGTGCTACAGATGCCCACGTGAAATCGTTGAGACAGTGTCTAAACTTGTCTACGATAACAAACTAAAAGCGGCGAAACCGAACTCAAGACAGTGTTACAAGACCATTGTGAACTTTGGTCCTGGAGACGTTGCTCATGAGGGACAATCTGCCTACAACGAAGCACAGTTGCGTTTCGCACTCGCATTTAGACAACAAAAGCGGTGGGATAACGTGACTTTCATATCTCCATATAATGCTATGAATGTGAAAGCATCCTTAGCAGGTTTCTCTACTCAGACCGTTGACTCTTCTCAAGGTTCTGAGTATGATTATGTTATCTTTTGCGTGACCACTGATTCAGCACACGCACTTAACATGGCTCGTTTGAACGTTGCCCTTACACGCGCAAAGATAGGTATCCTTGTGGTGTTTAGGCAGGCAAACGAACTTTACAATAGTTTGCAGTTTGAATCTATTGATTCACAGCTTCAGTCGAGTGCTGAGAAAAACCTCACACCACTGTTTAAGCGCTGCGGCTATGAGTATAATGGCGTCCATCCAGCTCATGCTTTGACCTGGCATGATTGTGGTGCAGAGTACCGCTGTGAGGAGCCACTTGCTAAATTAGTAGGAGTTGCCGATGGCACTCTTATATCATACAAAACCCTAGTATCCACACTTGGGTTTCTTCCATCACTTAAAATTGATGCATATCATAATATGTTCCTAACACGTGACGCGTGTCGCACCTATGTTCAGAGTTGGATCGGCATAGATGTTGAAGCAGCACACGCTATAAAACCTAACACCGGGACTAACCTGCCATTGCAAATAGGTTTTAGTACCGGAAAGAATTTTTCAGTCACTCCAGAGGGAATTTGGGTAAACGAGCACGGATCTTGCACTGAGCCCGTCCCTGCCAAAATACCTCCTGGAGAACAATTTCGTCACCTTAAAAAGGACATGCGCCAGGCGCGTCCTTGGAAGGTTGTTCGACGTGAGATTGCTACTCACATTGCTGAGGTAGCTCCTCATACTGATTATATATGCTTTGTCACTTGGGCTCACCAGCTTGAGCTAGCGACAATGCGCTACTTTGTCAAACTAGGTATGGAAGAGAAATGCTTTTGTGGCAGGCGAGCTTGTTTCACTAATGGAACTGAGTTCGCTTGCAAAGCACACCATTCTCTCACCATTCCACAATGTGATTATGTGTACAATCCATTCCTCATCGACGTGGCTACGTGGGGATTCTCGGGACGGCTTTCCACCAACCATGACGCGGTGTGCACATATCATGCTAATGCCCATGTTGCATCAGCTGATGCAATCATGACGGTATGTTTAGCTATCCATGAACTGTTCAGTACTGTTGACTGGAACCTTGAATTTCCAGTAACTGCTGAGCAATCGCAACTCAACAAGGCCTGTCGCTTAGTACAGGCAAATTACTTAAATATACTACTCACTACAACCAAAGCCACGGTGGTTCACGATATTGGTAACCCAAAAGGTATCCCTATCGTGCGCAAACCTGGTGTTAAATATCACTTCTATGATCAAGCACCCATTGTCAAACACGTTCAAAAACTAAAGTACAAGCCAGAGATGGAGGCCCGTTTCACCGATGGTTTGACTATGTTTTGGAATTGTAATGTTGACACATACCCTGCTAACGCCCTTGTGTGCCGCTACGACACTCATCGGCAGAAGCATTTAATTGGACCTAATGGTTCAGCACTATATGTTAATAAGCATGCTTTTCTCACCCCTGAAATGCATACTTATGCTACACATAAACTCAACTTGGCTCCACTCATCTACTACTCCACCACAGATTGTAGTAGTGAACAGCCTATTGTTGTTACCTACAGAGATTGTGTCACCCGGTGTAATACTGGAAAAACTCTCTGTCCAAACCATGCTCTTGAATACCAAGAGTTTATCAATGCATATAATCTCATGGCTCGCCATGGATTTAATGTTTATATACCACGCAATGTCAACATCTACAACTGTTGGCTTACTTTCACTAATCTCCAAAACCTTGAAAACTTAGCTTACAACTGTTATTATAAGAACTGCAATGCTCACGTTGATGGGCAGCTTGATGTAGTTATTAATAATAACGCTGTATATGCTAAGGTCGACAATAATCTTGTCAAACTTTTCGACAACCACACTAACTTACCTGTCTCAGTGGCTTTTGAACATTACACTAACAGGCATACCCGTTCACTGCCAACTACACAGCTGTTATCTGGTTTAGGCGTAACCGCCACCAGAAATTTCACTGTGTGGTTCGACAATGATACAATTTTCCAATACACTATTAATGTATCTACGTATACTGACATCGACCCTAGTACCCATGTTGTCCTCTGTGATGATAGGTACGGAACAGATTGGAGTCAGTTTAACCAACTTCCTAATGCAGTATTCCTCACCAAAACTAAGGTGAAGAAAACAGAACCGTTTGTTTGTACAGCACTGACCCTAAATGGCCTCGCCATTGACGGTGAAGAGCTATACATATATGTACGCTATAACAATCAACTGACCACATTTTCTACTACTTGTACACAGGGTAGAAATGTTGAGCAGTTTATACCTAAAACACCTATGGAAAGAGACTTCCTTGAGATGTCTCAGCAGTCCTTCATCCAGCAATATCAATTGCAGGAACTGGGTGTTGAACACATTATCTATGGTGATGATTCCAGTCCAGTTATTGGTGGAACTCACACACTTATCTCACTAGTTAAAAACAAGTTTGAACATCAGCTTGTCAACCATGTTTACAACCCAGTCCAGAACTGTGTTGTTACCTCACCTAACGCAAGCTCCAAGAACGTTTGCACTGTTCTTGATGTTCTTCTTGATGACTACATTGACATCATAAGACAAGCACATGCCAGTTACACAAGTAAATCTAAAGTATTCACTGTGTCAATTGACAATCAACAAATTAGATTCATGCTCTGGCATGATGAGCAAGTCAAGACTTGCTACCCAATCTTACAGTCACTTACCAATGGTTACCAGATGCCATCTGTGTACAAAACATTGGTTACTGACTTACAACCAGCCGACATCCCTAATTATCATTCCTACACCCCTCGGGTGCCTGGAGTAGTTAAGAATGTTATCAAGTACCGACAACTTTTCAACTACATAGTTAAAAAGGATAGGTTGGCAGTACCACACAATATGACCGTATTACACCTTGGGGCTGCATCTGCACTAGGTACAGCACCAGGTTCTTCAGTCATAAAACAAATGTTTCCTGAAGGAACTGTTCTTATTGACCTTGATATAAGAGAGTTCACTTCAGATGCTAACCAAATAATAGTTACAGACTACAGAACTTATATACCACCACACCACGTAGACGTCATATTTTCTGACCTCTACTGTTGTGATGACATACACTTCTTTGACAATCTAATAAGGATAGTTAAGGAGAGGCTCGCCCTCGGTGGTTCTATCTTTGTTAAGATAACTGAACATTCATTCTCACCCGAACTCTACTCACTTGCGGGTTGGTTCGATGATTATCAACTATTTTGCACAGCAGTTAATGCCTCGTCTTCAGAAGCATTTCTATGCTGTTTTAATTATTTGGGGCTTGCTAAGGAAAACATTAATGGTTTTAACTTACATGCTTCCTACATTCAATGGCGCAATGAAATAGCGTTGACACCAACCTATTCTCCTTTAGCGGACAACCCGGCTACTGCCTGTAAGCTAAAAGCAACGCCTATTATCTCGGCTCGTGAGTTAGAGAAGAAGCCTATTCTTCGCTATCTCGTTGCATCAGGACGCCTTCTTGTGAGGCCACCAGAATGCAGAGAGCTCTATTGATTATGACCTTACTTTGTCTCGTTCGAGCAAAGTTTGCTGATGATCTACTCGATTTGCTCACCTTCCCGGGTGCACATCGATTCTTACATAAACTCACGAGTAATTCCAGCAATCTCCACTCGCGGGCTAATAATTTTGATGTTGGCGTTCTTCCTGGCTACCCCACTAAGAACGTTAACCTCTTCTCACCACTTACTAACTCTACTTTGCCAATTAATGGCCTTCATCGGAGTTATCAACCACTCATGCTGAATTGTTTTACTAAAATAACTAACCACACTCTCAGCATGTATCTCCTACCTAGTGAGGTACAAACTTATAGCTGCGGCGGTGCTATGGTTAAATACCAGACACATGATGCAGTTCGTATCATTTTAGACCTCATTGCCACTGACCACATCTCTGTTGAAGTCGTTGGCCAACGTGGTGAAAATTATGTGTTTGTTTGTAGTGAGCAGTTTAACTATACCACTGCATTACATAACTCCACTTTCTTTTCACTTAATTCTGAGCTTTATTGCTTTACTAATAACACCTACTTAGGTATTCTTCCACCTGATTTAACTGACTTTACGGTCTACCGTACTGGTCAGTTCTATGCTAATGGTTACCTCTTAGGTACTTTACCTATTACTGTTAACTATGTCAGGTTGTATCGGGGTCAATTGGCTGCCAATAGTGTCCACTTTGCCCTAGCAAACCTAACCGATACACTCATAACACTTACCAATACCACTATATCGCAAATTACTTATTGTGATAAGTCAGTAGTTGACTCAATAGCATGCCAGCGCTCTTCTCATGAAGTGGAGGATGGGTTTTACTCCGACCCTAAATCTGCCGTTAGAGCTAGGCAACGTACTATTGTCACACTACCTAAGCTCCCCGAGCTTGAAGTAGTGCAGTTAAATATTTCTGCACACATGGATTTTGGCGAAGCCAGACTTGACAGCATTACCATCAATGGTAACACATCCTATTGTGTCACTAAGCCTTACTTCAGGCTTGAAACTAACTTTATGTGTACAGGTTGCACTATGAATCTGCGCACTGATACTTGTAGTTTTGACCTGTCAGCAGTAAATAATGGCATGTCATTCTCTCAATTCTGTCTAAGCACTGAATCTGGTGCTTGTGAGATGAAAATTATTGTTACCTACGTATGGAATTACTTGCTAAGGCAGCGTTTGTATGTTACTGCTGTAGAAGGCCAGACTCACACTGGAACCACTTCAGTACATGCAACAGACACTTCTAGTGTAATCACTGATGTCTGCACTGACTACACTATCTATGGAGTCTCTGGTACTGGCATTATTAAGCCATCAGATCTCTTATTGCACAATGGCATAGCATTCACCTCTCCAACAGGTGAGCTTTATGCATTTAAAAATATAACCACTGGCAAAACCCTCCAGGTCTTACCGTGTGAAACCCCTTCTCAACTGATTGTGATAAACAACACCGTCGTCGGTGCTATCACATCCAGTAACTCAGCTGAAAATAATAGGTTTACTACTACTATTGTCACACCTACTTTCTTTTATTCCACAAATGCCACCACTTTCAACTGCACTAAGCCTGTTTTGTCCTACGGACCCATCAGCGTGTGTAGTGATGGTGCAATTGTGGGAACATCCACATTACAGAATACTCGACCATCCATAGTTTCACTATATGATGGCGAAGTTGAAATACCATCTGCATTTTCTCTTTCTGTTCAGACGGAGTACTTGCAAGTTCAAGCAGAGCAAGTTATAGTTGATTGTCCTCAGTATGTATGCAATGGCAACAGCCGTTGTCTACAATTACTGGCACAATACACCTCAGCTTGCTCTAACATTGAAGCAGCTCTGCATTCCTCTGCACAGTTGGATAGCAGAGAGATTATAAATATGTTTCAAACATCAACACAGTCCTTGCAGTTGGCTAATATTACCAACTTCAAGGGTGACTACAATTTTAGCAGCATAATAACCCCCAGAATTGGTGGCAGATCTGCTATTGAAGACCTTCTTTTTAATAAAGTTGTTACTAGTGGCCTTGGCACTGTTGATCAGGACTACAAAGCCTGCTCTAGAGACATGGCCATCGCTGACTTAGTTTGTTCCCAGTATTACAATGGCATCATGGTTCTACCTGGTGTTGTTGATGCTGAGAAAATGGCAATGTATACTGGCTCTCTTACTGGAGCTATGGTATTTGGGGGACTGACTGCTGCAGCGGCAATACCATTCGCCACGGCAGTACAAGCCCGCCTCAATTATGTCGCACTGCAAACAAATGTACTACAAGAAAACCAGAAAATACTTGCAGAATCATTTAACCAAGCAGTTGGCAATATATCACTTGCACTATCTTCTGTTAATGATGCCATCCAGCAAACTTCTGAGGCTCTTAACACCGTAGCTATTGCTATTAAAAAGATTCAAACAGTTGTCAACCAGCAGGGTGAGGCATTATCACACCTGACTGCACAGCTGTCAAACAATTTTCAAGCAATTTCGACTTCTATTCAAGACATTTACAACCGTCTTGAGGAAGTAGAGGCTAACCAGCAAGTTGACCGTCTCATTACAGGACGGTTGGCTGCACTTAATGCATATGTTACTCAGTTACTCAATCAGATGTCTCAGATTAGACAATCTCGATTGTTAGCTCAGCAAAAGATTAATGAGTGTGTCAAATCTCAGTCGTCCAGATACGGTTTCTGTGGAAATGGCACACACATCTTCTCACTTACACAGACTGCACCAAATGGCATATTTTTCATGCATGCAGTACTTGTACCCAACAAATTCACACGTGTCAACGCTTCTGCGGGTATTTGTGTGGATAATACGAGAGGCTACTCATTGCAGCCTCAACTTATACTCTACCAGTTTAATAACTCCTGGAGAGTTACACCTAGAAATATGTATGAACCCAGACTGCCCCGGCAAGCTGATTTCATACAATTAACTGATTGCAGCGTTAATTTTTACAATACCACCGCTGCTAATCTTCCCAATATTATCCCTGACGTTATAGATGTCAATCAAACAGTCAGTGACATTATTGACAATTTACCTACAGCAACACCTCCTCAGTGGGATGTTGGTATTTATAACAACACTATTCTCAACCTCACCGTTGAGATTAATGATCTACAAGAGCGGTCTAAAAACCTCTCACAGATTGCAGATCGTTTACAAAATTATATTGACAATCTTAACAATACTCTAGTTGACCTTGAATGGCTCAACAAAGTGGAAACTTACCTTAAATGGCCGTGGTATGTATGGCTTGCCATAGCCCTGGCTCTTATTGCATTTGTGACAATCCTCATAACAATCTTTCTCTGTACTGGTTGTTGTGGTGGTTGTTTTGGTTGTTGTGGCGGTTGTTTTGGCCTTTTCTCTAAGAAGAAAAGGTATACCGACGACCAACCAACACCGTCCTTTAAGTTTAAGGAATGGTAGTCGACGACTGGGCCGTTACCATCCCTGGACAATATATTATTGCTATACTAGTTGTCATCTGCATTGGTGTGGCACTACTTTTTATTAACACTTGCTTAGCTTGTGTTAAATTATTTTACAAGTGCTACCTAGGGGCAGCATATCTTGTTAGGCCTATTATAGTGTACTACTCCAAGCCGAACCCCGTACCTGAGGATGAGTTTGTAAAAGTACACCAATTTCCTAGAAACACTCACTATGTCTGACGCAGAAGAGTGGCAAATTATTGTTTTCATTGCGATCATATGGGCGCTTGGCGTCATCCTCCAAGGAGGCTATGCCACGCGTAACCGTGTGATCTATGTTATTAAACTTATTCTGCTTTGGCTGCTCCAACCCTTCACCCTAGTGGTGACCATTTGGACCGCAGTTGACAGATCATCTAAGAAGGACGCAGTTTTCATTGTGTCCATAATTTTTTCCGTACTGACCTTCATATCCTGGGTCAAGTACTGGTATGACTCAATTCGCTTATTAATGAAAACCAGATCTGCATGGGCACTCTCACCTGAGAGTAGACTCCTTGCAGGGATTATGGATCCAATGGGTACATGGAGGTGCATTCCCATCGACCACATGGCTCCAATTCTCACACCAGTCGTTAAGCATGGCAAGCTCAAGCTACATGGGCAAGAGCTGGCCAATGGCATATCAGTTAGAAATCCGCCACAGGATATGGTGATAGTGTCACCAAGTGACACCTTTCACTACACTTTTAAGAAACCTGTGGAATCAAACAGCGATCCAGAATTCGCTGTTCTGATATACCAGGGTGACCGCGCTTCAAACGCTGGACTTCACACCATAACCACTTCAAAGGCCGGTGACGCTCGCCTGTATAAGTATATGTAATGTGCAACTGCCATCTGCAGCTGCGAGATTTATATAGATTGTGCAATAAGCTGCACATCAGAAGAGACGATGTTCCTGAGCTTATTGACCCTCTCGTTAAAACTCGCTGTTTTGCTTACAGTCTCGTGGTTCTTGCTAATGCTAATCCAATTGCATTTAGCATACTACCTCGGAAAATTCTTATCAATGGTGAGCCTTTACTGCTTGAATATGGTAGCATATATGGTAAAGACTTTATCATTAGACCATCGCTCCAAGTCATTCTTGAAGATGAATTAAATTAAAGTTTTGACACCAATCTATCATGGCTGCACCAGTGGTCCCTACTACTGACGCGTCTTGGTTTCAGGTGCTCAAAGCTCAAAATAAAAAGGCTACTCATCCTCAGTTTCGTGGCAATGGAGTTCCGCTTAACTCCGCCATCAAACCCGTTGAAAACCATGGCTACTGGCTGCGTTACACCAGACAAAAGCCAGGTGGTACTCCGATTCCTCCATCCTATGCCTTTTATTATACTGGCACAGGTCCCAGAGGAAATCTTAAGTATGGTGAACTCCCTCCTAATGATACCCCAGCAACCACTCGTGTTACTTGGGTTAAGGGTGCGGGAGCTGACACTTCTATTAAACCTCATGTTGCCAAACGCAACCCCAACAATCCTAAACATCAGCTGCTACCTCTCCGATTCCCAACCGGAGATGGCCCAGCTCAAGGTTTCAGAGTTGACCCCTTCAACGCTAGAGGAAGACCTCAGGAGCGTGGAAGTGGCCCAAGATCTCAATCTGTTAACTCCAGAGGCACAGGCAATCAGCCCAGGAAACGCGACCAATCTGCACCCGCTGCAGTACGTCGTAAGACCCAACATCAAGCTCCCAAGCGGACTTTACCCAAGGGTAAAACCATTTCTCAGGTATTTGGCAACCGGTCTCGTACTGGTGCCAATGTCGGCTCTGCAGACACTGAGAAGACGGGTATGGCTGATCCTCGCATCATGGCTCTAGCCAGACATGTGCCTGGTGTTCAGGAAATGCTTTTCGCTGGTCACCTTGAGAGCAACTTTCAGGCAGGGGCAATTACCCTTACCTTCTCATACTCAATCACAGTCAAGGAGGGTTCTCCTGACTATGAGAGACTTAAGGATGCGCTCAATACGGTCGTTAACCAGACCTATGAGCCACCCACTAAACCAACTAAGGACAAGAAGCCTGACAAACAAGACCAGTCTGCTAAACCCAAACAGCAGAAGAAACCTAAAAAGGTAACTCTGCCAGCAGACAAACAGGATTGGGAGTGGGATGATGCTTTTGAGATAAAGCAGGAATCAGCAGCGTAGACATTTCTCTACCCACCCAACTCCACTCAAATATCTCTTTGATTCCAGAGAGTCATAGTGTATAGCCAGAGAGCCAGTCAGAGGGCGCTATCATGCAAACTAGGGCTGGCTACTCTAGCACAGAATCACATCCCGATAATCAACAGTGCTAGAAGGTTGATTATACCATTTAATATGCCGAGGCCACGCGGAGTACGATCGAGGGTACAGCATAATCTCAACTTTTGTTGAGCCACAATTTTAATCCTAATTGGAGAAGGCCAAAGGACTGTACTACTTTTGTAGGTGTAGCAGTCGCCCAGTGGGAAAGCGCCAACTAGGTTACAATTGTGGTGGGGACAAATTAGGGGAAATTAAATTGG

CTTATAGGGGGGATGGAGCA
