# Supplementary material for: Phylogenetically evolutionary analysis provides insights into the genetic diversity and adaptive evolution of porcine deltacoronavirus
Source: BMC Vet Res. 2024 Jan 10;20:22. doi: 10.1186/s12917-023-03863-2 (PMC10782762; doi:10.1186/s12917-023-03863-2)
Supplement: Supplementary file 4 — Supplementary Material 4: Supplementary file 2. The original full-length gels for the PDCoV laboratory diagnosis [file 12917_2023_3863_MOESM4_ESM.pdf]

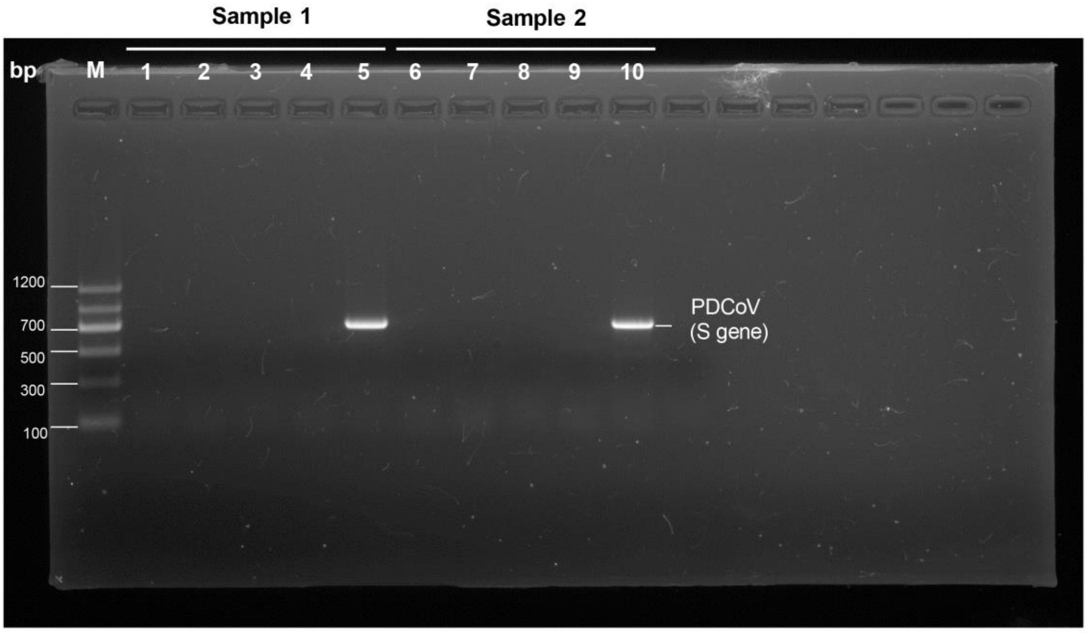

Supplementary file 2. The original full-length gels for the PDCoV laboratory diagnosis. The PCR result of nucleic acids testing of the potential viral pathogens. PEDV (line 1 and 6), TGEV (line 2 and 7), PoRV (line 3 and 8), SADS-CoV (line 4 and 9), and PDCoV (line 5 and 10).
