# Supplementary material for: Phylogenetically evolutionary analysis provides insights into the genetic diversity and adaptive evolution of porcine deltacoronavirus
Source: BMC Vet Res. 2024 Jan 10;20:22. doi: 10.1186/s12917-023-03863-2 (PMC10782762; doi:10.1186/s12917-023-03863-2)
Supplement: Supplementary file 5 — Supplementary Material 5: Supplementary file 3. The raw data of positive selection analysis across the spike gene [file 12917_2023_3863_MOESM5_ESM.pdf]

Supplementary file 3. The raw data of positive selection analysis across the spike gene.

Positive selection analysis across the spike gene using Fast Unconstrained Bayesian AppRoximation (FUBAR)

Site	Partition	α	β	β-α	Prob[α>β]	Prob[α<β]	BayesFactor [α<β]
40	1	0.887	9.828	8.94	0.002	0.971	81.673
44	1	0.704	12.024	11.32	0	0.988	206.233
46	1	0.624	6.088	5.464	0.009	0.974	92.586
62	1	0.589	5.268	4.679	0.007	0.976	97.181
110	1	1.246	10.887	9.641	0.007	0.941	39.089
111	1	1.348	8.045	6.697	0.01	0.921	28.414
123	1	0.619	7.392	6.773	0.001	0.989	223.411
136	1	0.747	7.449	6.702	0.001	0.98	122.615
137	1	0.588	6.224	5.636	0.003	0.986	167.823
149	1	0.596	16.64	16.044	0	0.998	1309.25
169	1	0.53	14.193	13.663	0	0.998	1522.376
178	1	1.315	9.354	8.039	0.01	0.928	31.54
183	1	0.742	7.342	6.6	0.001	0.981	123.744
397	1	0.793	10.019	9.226	0.001	0.979	114.466
630	1	0.999	10.137	9.138	0.003	0.961	60.921
642	1	1.632	40.41	38.779	0.001	0.992	314.641
698	1	0.583	4.435	3.852	0.022	0.954	50.205
798	1	0.841	6.548	5.707	0.012	0.954	50.675
838	1	0.826	4.983	4.156	0.047	0.909	24.301
1086	1	1.541	11.423	9.882	0.014	0.923	29.056
1090	1	1.346	11.021	9.675	0.009	0.934	34.546

Positive selection analysis across the spike gene using Fixed Effects Likelihood (FEL)

Partition	codon	alpha	beta	alpha=beta	LRT	p-value	Total branch length	p-asmp	class
1	38	1.365	4.311	3.174	2.727	**0.0987**	3.159	**0.0000**	Diversifying
1	40	0.000	3.357	2.582	3.671	**0.0554**	2.570	**0.0000**	Diversifying
1	44	0.000	4.020	2.959	4.965	**0.0259**	2.945	**0.0000**	Diversifying
1	46	0.000	2.363	1.359	4.391	**0.0361**	1.352	**0.0000**	Diversifying
1	62	0.000	2.062	1.253	4.960	**0.0259**	1.248	**0.0000**	Diversifying
1	110	0.000	3.542	3.035	2.718	**0.0992**	3.021	**0.0000**	Diversifying
1	111	0.000	3.420	2.768	2.893	**0.0890**	2.755	**0.0000**	Diversifying
1	123	0.000	2.928	1.694	5.464	**0.0194**	1.686	**0.0000**	Diversifying
1	136	0.000	3.072	2.042	4.836	**0.0279**	2.032	**0.0000**	Diversifying
1	137	0.000	2.286	1.331	5.371	**0.0205**	1.325	**0.0000**	Diversifying
1	140	0.000	1.376	0.868	2.769	**0.0961**	0.864	**0.0000**	Diversifying
1	149	0.000	6.284	4.552	7.765	**0.0053**	4.531	**0.0000**	Diversifying
1	169	0.000	4.853	2.804	8.687	**0.0032**	2.791	**0.0000**	Diversifying
1	183	0.000	3.022	2.005	4.884	**0.0271**	1.996	**0.0000**	Diversifying
1	397	0.000	3.603	2.575	4.104	**0.0428**	2.563	**0.0000**	Diversifying
1	630	0.000	4.148	3.167	4.770	**0.0290**	3.152	**0.0000**	Diversifying
1	642	0.000	12.050	10.165	4.084	**0.0433**	10.117	**0.0000**	Diversifying
1	698	0.000	1.784	1.052	4.227	**0.0398**	1.047	**0.0000**	Diversifying

Positive selection analysis across the spike gene using Mixed Effects Model of Evolution (MEME)

Site	Partition	α	β-	p-	β+	p+	LRT	p-value	# branches under selection	Total branch length	MEME LogL	FEL LogL
40	1	0.00	0.00	0.00	3.37	1.00	3.67	0.08	2.00	0.00	-43.59	-43.59
42	1	0.56	0.23	0.97	44.43	0.03	4.47	0.05	1.00	0.00	-26.04	-24.00
44	1	0.00	0.00	0.01	4.08	0.99	4.97	0.04	4.00	0.00	-48.33	-48.33
46	1	0.00	0.00	0.00	2.34	1.00	4.39	0.05	3.00	0.00	-25.33	-25.33
62	1	0.00	0.00	0.00	2.05	1.00	4.96	0.04	1.00	0.00	-35.35	-35.35
123	1	0.00	0.00	0.00	2.95	1.00	5.47	0.03	3.00	0.00	-28.56	-28.56
136	1	0.00	0.00	0.01	3.08	0.99	4.84	0.04	2.00	0.00	-37.16	-37.16
137	1	0.00	0.00	0.85	18.87	0.15	5.63	0.03	5.00	0.00	-33.75	-33.62
146	1	0.00	0.00	0.99	93.08	0.01	9.53	0.00	1.00	0.00	-15.47	-11.50
149	1	0.01	0.00	0.47	12.11	0.53	7.96	0.01	2.00	0.00	-61.09	-60.99
169	1	0.00	0.00	0.53	10.93	0.47	8.79	0.01	8.00	0.00	-52.54	-52.49
183	1	0.00	0.00	0.01	3.03	0.99	4.88	0.04	4.00	0.00	-32.00	-32.00
317	1	0.00	0.00	0.99	922.30	0.01	6.78	0.02	2.00	0.00	-23.97	-20.87
397	1	0.00	0.00	0.01	3.70	0.99	4.11	0.06	6.00	0.00	-43.38	-43.38
489	1	0.00	0.00	0.96	240.65	0.04	6.61	0.02	5.00	0.00	-41.93	-39.47
508	1	0.00	0.00	0.99	386.14	0.01	3.60	0.08	2.00	0.00	-21.10	-19.57
557	1	0.00	0.00	0.97	24.89	0.03	5.33	0.03	1.00	0.00	-15.77	-13.53
563	1	0.00	0.00	0.99	80.08	0.01	5.90	0.02	1.00	0.00	-17.04	-14.09
564	1	2.03	0.00	0.99	73.40	0.01	4.59	0.05	1.00	0.00	-25.63	-22.11
565	1	0.00	0.00	0.99	1255.99	0.01	10.68	0.00	1.00	0.00	-20.38	-14.06
571	1	0.00	0.00	0.00	3.60	1.00	5.58	0.03	1.00	0.00	-37.04	-37.04
590	1	0.00	0.00	0.97	187.81	0.03	3.91	0.07	1.00	0.00	-27.62	-26.45
630	1	0.00	0.00	0.81	26.30	0.19	7.41	0.01	8.00	0.00	-48.21	-46.89
642	1	0.00	0.00	0.41	21.37	0.59	4.42	0.05	8.00	0.00	-92.68	-92.41
698	1	0.00	0.00	0.01	1.81	0.99	4.23	0.06	2.00	0.00	-30.28	-30.28
798	1	0.00	0.00	0.95	80.63	0.05	3.95	0.06	4.00	0.00	-31.88	-31.21
818	1	0.00	0.00	0.96	44.46	0.04	5.61	0.03	2.00	0.00	-22.57	-20.24
867	1	0.00	0.00	0.91	23.28	0.09	3.68	0.07	2.00	0.00	-25.32	-24.11
876	1	0.00	0.00	0.98	206.30	0.02	11.47	0.00	2.00	0.00	-23.97	-19.32
